# Structured lifestyle education for people with schizophrenia, schizoaffective disorder and first-episode psychosis (STEPWISE): randomised controlled trial

**DOI:** 10.1192/bjp.2018.167

**Published:** 2019-02

**Authors:** Richard I. G. Holt, Rebecca Gossage-Worrall, Daniel Hind, Michael J. Bradburn, Paul McCrone, Tiyi Morris, Charlotte Edwardson, Katharine Barnard, Marian E. Carey, Melanie J. Davies, Chris M. Dickens, Yvonne Doherty, Angela Etherington, Paul French, Fiona Gaughran, Kathryn E. Greenwood, Sridevi Kalidindi, Kamlesh Khunti, Richard Laugharne, John Pendlebury, Shanaya Rathod, David Saxon, David Shiers, Najma Siddiqi, Elizabeth A. Swaby, Glenn Waller, Stephen Wright

**Affiliations:** 1 Professor in Diabetes and Endocrinology, Human Development and Health Academic Unit, Faculty of Medicine, University of Southampton and Honorary Consultant Physician, Division B, University Hospital Southampton NHS Foundation Trust, UK; 2 Trial Manager (Research Associate), Clinical Trials Research Unit, School of Health and Related Research, University of Sheffield, UK; 3 Reader in Complex Interventions and Assistant Director, Clinical Trials Research Unit, School of Health and Related Research, University of Sheffield, UK; 4 Senior Medical Statistician, Clinical Trials Research Unit, School of Health and Related Research, University of Sheffield, UK; 5 Professor of Health Economics and Director of King's Health Economics, Institute of Psychiatry, Psychology & Neuroscience, King's College London, UK; 6 Research Assistant, Institute of Psychiatry, Psychology & Neuroscience, King's College London, UK; 7 Associate Professor in Physical Activity, Sedentary Behaviour and Health, Diabetes Research Centre, University of Leicester, UK; 8 Health Psychologist and Visiting Professor, Faculty of Health and Social Science, University of Bournemouth, UK; 9 Director: Structured Education Research Portfolio, Leicester Diabetes Centre, University Hospitals of Leicester and Honorary Associate Professor, Diabetes Research Centre, University of Leicester, UK; 10 Professor of Diabetes Medicine, Diabetes Research Centre, University of Leicester, UK; 11 Professor of Psychological Medicine, Institute of Health Research, University of Exeter Medical School, UK; 12 Consultant Clinical Psychologist and Senior Research Associate, Leicester Diabetes Centre, University Hospitals of Leicester, UK; 13 Patient Representative, Independent Service User Consultant, UK; 14 Associate Director, Psychosis Research Unit, Greater Manchester Mental Health NHS Foundation Trust, UK; 15 Reader, Institute of Psychiatry, Psychology & Neuroscience, King's College London and Consultant Psychiatrist and Director of Research, National Psychosis Service, South London and Maudsley NHS Foundation Trust, UK; 16 Consultant Clinical Psychologist, Sussex Partnership NHS Foundation Trust and Professor in Clinical Psychology, Sussex Psychosis Research Interest Group, School of Psychology, University of Sussex, UK; 17 Consultant Psychiatrist, Rehabilitation and Recovery, South London and Maudsley NHS Foundation Trust and Senior Clinical Lecturer, Institute of Psychiatry, Psychology & Neuroscience, King's College London, UK; 18 Professor of Primary Care Diabetes and Vascular Medicine, Diabetes Research Centre, University of Leicester, UK; 19 Consultant Psychiatrist and Honorary Senior Lecturer, Cornwall Partnership NHS Foundation Trust, UK; 20 Retired NHS Community Psychiatric Nurse, UK; 21 Consultant Psychiatrist and Director of Research, Southern Health NHS Foundation Trust and Visiting Professor, Faculty of Science, University of Portsmouth, UK; 22 Research Fellow, Mental Health Unit, School of Health and Related Research, University of Sheffield, UK; 23 Honorary Research Consultant, Psychosis Research Unit, Greater Manchester Mental Health NHS Foundation Trust and Honorary Reader in Early Psychosis, School of Health Sciences, Division of Psychology and Mental Health, University of Manchester, UK; 24 Clinical Senior Lecturer in Psychiatry, Health Sciences, University of York, Hull York Medical School and Consultant Psychiatrist, Bradford District Care NHS Foundation Trust, UK; 25 Study Manager, Clinical Trials Research Unit, School of Health and Related Research, University of Sheffield, UK; 26 Professor of Psychology, Department of Psychology, University of Sheffield, UK; 27 Lead Consultant, Early Intervention Psychiatry, Tees Esk & Wear Valleys NHS Foundation Trust, UK

**Keywords:** Schizophrenia, psychosis, antipsychotic, obesity, overweight, exercise, healthy diet, lifestyle, cost benefit analysis

## Abstract

**Background:**

Obesity is a major challenge for people with schizophrenia.

**Aims:**

We assessed whether STEPWISE, a theory-based, group structured lifestyle education programme could support weight reduction in people with schizophrenia.

**Method:**

In this randomised controlled trial (study registration: ISRCTN19447796), we recruited adults with schizophrenia, schizoaffective disorder or first-episode psychosis from ten mental health organisations in England. Participants were randomly allocated to the STEPWISE intervention or treatment as usual. The 12-month intervention comprised four 2.5 h weekly group sessions, followed by 2-weekly maintenance contact and group sessions at 4, 7 and 10 months. The primary outcome was weight change after 12 months. Key secondary outcomes included diet, physical activity, biomedical measures and patient-related outcome measures. Cost-effectiveness was assessed and a mixed-methods process evaluation was included.

**Results:**

Between 10 March 2015 and 31 March 2016, we recruited 414 people (intervention 208, usual care 206) with 341 (84.4%) participants completing the trial. At 12 months, weight reduction did not differ between groups (mean difference 0.0 kg, 95% CI −1.6 to 1.7, *P* = 0.963); physical activity, dietary intake and biochemical measures were unchanged. STEPWISE was well-received by participants and facilitators. The healthcare perspective incremental cost-effectiveness ratio was £246 921 per quality-adjusted life-year gained.

**Conclusions:**

Participants were successfully recruited and retained, indicating a strong interest in weight interventions; however, the STEPWISE intervention was neither clinically nor cost-effective. Further research is needed to determine how to manage overweight and obesity in people with schizophrenia.

**Declaration of interest:**

R.I.G.H. received fees for lecturing, consultancy work and attendance at conferences from the following: Boehringer Ingelheim, Eli Lilly, Janssen, Lundbeck, Novo Nordisk, Novartis, Otsuka, Sanofi, Sunovion, Takeda, MSD. M.J.D. reports personal fees from Novo Nordisk, Sanofi-Aventis, Lilly, Merck Sharp & Dohme, Boehringer Ingelheim, AstraZeneca, Janssen, Servier, Mitsubishi Tanabe Pharma Corporation, Takeda Pharmaceuticals International Inc.; and, grants from Novo Nordisk, Sanofi-Aventis, Lilly, Boehringer Ingelheim, Janssen. K.K. has received fees for consultancy and speaker for Novartis, Novo Nordisk, Sanofi-Aventis, Lilly, Servier and Merck Sharp & Dohme. He has received grants in support of investigator and investigator-initiated trials from Novartis, Novo Nordisk, Sanofi-Aventis, Lilly, Pfizer, Boehringer Ingelheim and Merck Sharp & Dohme. K.K. has received funds for research, honoraria for speaking at meetings and has served on advisory boards for Lilly, Sanofi-Aventis, Merck Sharp & Dohme and Novo Nordisk. D.Sh. is expert advisor to the NICE Centre for guidelines; board member of the National Collaborating Centre for Mental Health (NCCMH); clinical advisor (paid consultancy basis) to National Clinical Audit of Psychosis (NCAP); views are personal and not those of NICE, NCCMH or NCAP. J.P. received personal fees for involvement in the study from a National Institute for Health Research (NIHR) grant. M.E.C. and Y.D. report grants from NIHR Health Technology Assessment, during the conduct of the study; and The Leicester Diabetes Centre, an organisation (employer) jointly hosted by an NHS Hospital Trust and the University of Leicester and who is holder (through the University of Leicester) of the copyright of the STEPWISE programme and of the DESMOND suite of programmes, training and intervention fidelity framework that were used in this study. S.R. has received honorarium from Lundbeck for lecturing. F.G. reports personal fees from Otsuka and Lundbeck, personal fees and non-financial support from Sunovion, outside the submitted work; and has a family member with professional links to Lilly and GSK, including shares. F.G. is in part funded by the National Institute for Health Research Collaboration for Leadership in Applied Health Research & Care Funding scheme, by the Maudsley Charity and by the Stanley Medical Research Institute and is supported by the by the Biomedical Research Centre at South London and Maudsley NHS Foundation Trust and King's College London.

People with schizophrenia die 10–20 years earlier than the general population, with approximately 75% of deaths resulting from physical illness.^1^ The twofold increased prevalence of overweight and obesity contributes to this excess mortality.^2^ Some, but not all, studies suggest that dietary and physical activity interventions may reduce weight gain.^3^^–^^7^

Many weight loss programmes involve one-to-one strategies to promote behaviour change but these are unlikely to be affordable in many healthcare settings.^8^ Group-based structured education offers an alternative approach,^9^ and has been adopted by the UK National Health Service (NHS) Diabetes Prevention Programme.^10^ The National Institute for Health and Care Excellence (NICE) recommends that lifestyle interventions should be offered to people taking antipsychotics but there is insufficient evidence to inform how these should be commissioned.^11^

We designed the STEPWISE group-based lifestyle structured education and then conducted a randomised controlled trial (RCT) to evaluate whether STEPWISE could lead to clinically relevant weight loss after a year in adults with schizophrenia, schizoaffective disorder or first-episode psychosis. Further objectives were to assess the impact on physical activity, diet, biomedical measures and quality of life, intervention fidelity, acceptability to participants and mental health services, and cost-effectiveness.

## Method

### Study design

STEPWISE was a two-arm, parallel group RCT comparing the STEPWISE intervention with treatment as usual (TAU) (study registration: ISRCTN19447796). The study took place in ten English NHS mental health trusts in urban and rural locations. The trial was approved by UK National Research Ethics Committee, Yorkshire & the Humber - South Yorkshire, (reference 14/YH/0019) and conducted in accordance with Good Clinical Practice. The trial protocol has been reported.^12^

### Participants

Researchers at each site worked with local mental health clinicians to identify potentially eligible patients from clinic lists and case notes. We used posters and leaflets to encourage self-referral. We recruited adults (≥18 years) with a clinical diagnosis of schizophrenia, schizoaffective disorder (ICD-10 codes F20, F25) or first-episode psychosis (defined as <3 years since presentation to mental health services).^13^ The Operational Criteria Checklist (OPCRIT+) was completed using case-note review to assess whether the clinical diagnosis matched an objective measure of psychiatric illness.^14^

All participants had been prescribed an antipsychotic for ≥1 month and were able and willing to participate in a group education programme. Participants had a body mass index (BMI) ≥25 kg/m^2^ (≥23 kg/m^2^ for South Asian and Chinese backgrounds) or expressed concern about their weight.

People were excluded if they had a physical illness that could seriously reduce their life expectancy or ability to participate, that would independently have an impact on metabolic measures and weight, for example Cushing syndrome, or were currently pregnant or less than 6 months postpartum. High levels of psychiatric symptoms, as judged by the principal investigator, which could seriously affect participation and ability to put into practice the learning from the intervention sessions were a further exclusion criterion. People with significant alcohol or substance misuse, a primary diagnosis of psychotic depression, mania or intellectual disability (also known as learning disability in UK health services) were excluded. People currently (or within the past 3 months) engaged in a weight-management programme or unable to speak and read English were also excluded. Participants provided written informed consent before trial entry.

### Randomisation and masking

The Sheffield Clinical Trials Research Unit generated a computerised randomisation list using permuted blocks of random sizes to allocate participants to either TAU plus the STEPWISE intervention or TAU alone in a 1:1 ratio, stratified by site and time since antipsychotic initiation (<3 months or ≥3 months). After randomisation, an unmasked researcher informed the participant and their general practitioner of the allocation. Research outcome assessors were masked to treatment allocation. Breaks or suspected breaks in masking were recorded. The nature of the intervention meant that participants were not masked.

### Interventions

#### STEPWISE structured education lifestyle programme

We developed the STEPWISE intervention using the Medical Research Council framework for complex interventions (Supplementary Appendix 1 available at https://doi.org/10.1192/bjp.2018.167). Following a systematic literature review, a team with expertise in obesity, lifestyle interventions, behaviour change and mental health and people with schizophrenia designed the prototype intervention, which was piloted and amended in four iterative cycles.

**Fig. 1.**
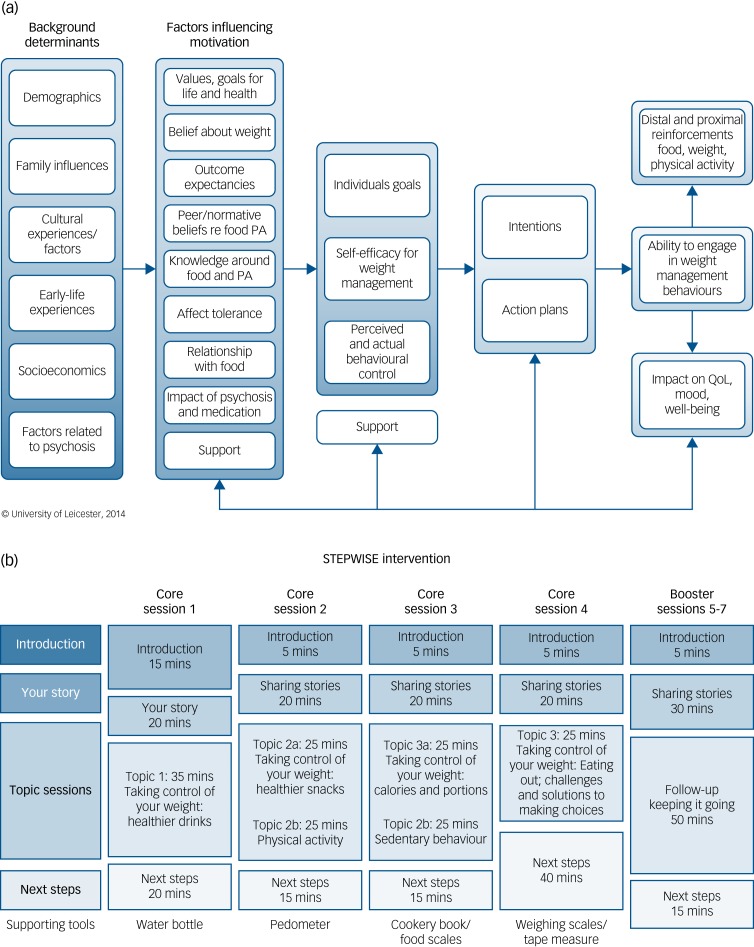
The STEPWISE intervention. (a) Theoretical framework. The STEPWISE intervention was codesigned by a team with expertise in the development of obesity and lifestyle intervention programmes, mental healthcare professionals and researchers, and service users and refined during a four-cycle pilot. It was underpinned by self-regulation and self-efficacy theories and the relapse prevention model. (b). Curriculum. The STEPWISE intervention comprised four 2.5 h foundation group education sessions, designed to be delivered to small groups of 6–8 participants over 4 consecutive weeks followed by three 2.5 h follow-up ‘booster’ sessions at 3-monthly intervals and fortnightly support, usually by telephone. The content was determined by the specific difficulties described by people with schizophrenia. The sessions incorporated adequate breaks. The educational style was non-judgemental and facilitative to allow the participants to discuss their beliefs about weight and explore own solutions. Strategies was employed to maintain engagement including telephone call reminders, provision of taxis to the venue, afternoon sessions with lunch provided and use of incentives described as supporting tools. PA, physical activity; QoL, quality of life.

We considered three areas that are core to weight-management interventions in people with schizophrenia when developing the theoretical framework that guided the intervention (Fig. 1(a)):

(a) behaviour change theory specifically with a focus on food and physical activity;
(b) psychological processes underlying weight management;
(c) challenges of living with psychosis and its impact on eating and weight.

Based on established psychological theories, appropriate behaviour change techniques were used to address problem behaviours.

STEPWISE took place over approximately 12 months. Groups of participants (median 6, range 3–11) attended a foundation course of four weekly 2.5-hour group sessions delivered by two trained facilitators (Fig. 1(b)). This was followed by 1:1 support contact, mostly by telephone, lasting about 10 min, approximately every 2 weeks for the remainder of the intervention period. A trained facilitator carried out the support contact to promote behaviour change and continued engagement. Further 2.5-hour group-based booster sessions took place at approximately 4, 7 and 10 months after randomisation giving a total intervention duration of ~25.5 h.

All sessions started at lunchtime with the provision of a healthy lunch. After an initial introduction, participants were invited to ‘share their story’. This provided the facilitators with feedback on what changes the person had made and what remained challenging. The facilitators used a non-judgemental style to encourage openness, problem-solving and sharing successful strategies. Specific changes and challenges were recorded so that the participants could refer back to their individualised solutions.

The next part was entitled ‘Taking control of your weight’ to reinforce the focus of the intervention. Each session covered one or two aspects of how lifestyle changes could help the participants take control of their weight. Four topics covered diet whereas two focused on physical activity. A facilitative approach, as opposed to a didactic teaching style, was used to enable participants to discuss their beliefs about weight and explore their own solutions.

The final section was devoted to action planning, when the participants developed their own individualised goals and solutions. As the participants departed, they were given supporting tools to reinforce the key messages of the session.

Each centre had four to six trained facilitators to maintain consistency across sessions and support contact. We recorded intervention attendance and level of support contact. We invited participants to complete an anonymous six-question ‘session feedback’ form at the end of each session (supplementary Appendix 2a).

#### Control arm

As no consistent lifestyle education programme was offered across sites,^1^,^5^ we provided printed advice on lifestyle and the risks associated with weight gain for all participants. We recorded whether participants attended other weight-management or physical activity programmes outside the trial.

### Outcomes

Trial assessments were undertaken at the participant's home or mental health organisation, after consent but before randomisation and at 3 and 12 months post-randomisation (supplementary Appendix 3).

The primary end-point was weight change at 12 months after randomisation. A medical and psychiatric history, including smoking and current medication, was obtained. Height (baseline only), weight, waist circumference and blood pressure were measured (supplementary Appendix 2b). Participants wore a wrist GENEActiv accelerometer for 7 days to assess physical activity (mean acceleration and mean time spent in moderate-to-vigorous physical activity) (supplementary Appendix 2c).

Research staff helped participants complete the self-report questionnaires by reading the questions, checking understanding and providing available answer options. We assessed dietary intake with the Adapted Dietary Instrument for Nutrition Education questionnaire.^1^,^6^ We used questionnaires to assess patient-reported outcome measures, including quality of life (RAND SF-36 and EQ-5D-5L),^17^^,^^18^ health beliefs (adapted Brief Illness Perception Questionnaire),^1^,^9^ psychiatric symptoms (Brief Psychiatric Rating Scale)^20^ and depressive symptoms (9-item Patient Health Questionnaire).^2^,^1^

Assessments of fasting glucose, glycated haemoglobin (HbA_1c_) and lipid profile were made at baseline and 12 months post-randomisation.

### Safety assessments

We monitored adverse events at 3 and 12 months. Expected serious adverse events included psychiatric hospital admissions, self-harm, suicide attempt and death from suicide. An independent data monitoring committee and trial steering committee oversaw the conduct and safety of the trial.

### Cost-effectiveness analysis

We undertook an economic evaluation from a health and social care and societal perspective. Health and social care costs included the costs of medicines and NHS professionals in primary and community care and in-patient settings, and social care costs. Societal costs were calculated using police costs, productivity losses from lost education and employment and informal care costs. The intervention cost was based on staff time plus overheads and included training and supervision. The Client Service Receipt Inventory was used to record service use.^22^ Costs were calculated using appropriate unit cost information. Cost-effectiveness was assessed by combining cost with the primary outcome and quality-adjusted life-years (QALY) generated from the EQ-5D-5L questionnaire. We constructed incremental cost-effectiveness ratios to demonstrate the cost per extra QALY gained and uncertainty around estimates was explored using cost-effectiveness planes and acceptability curves.

### Process evaluation

We undertook a process evaluation using a published framework and a logic model that focused on resources, activities and process outcomes (reach, delivery, fidelity and receipt of intervention).^23^ Qualitative data were collected via semi-structured telephone interviews with participants (*n* = 24), intervention facilitators (*n* = 20) and intervention developers (*n* = 7). The interviews were recorded and coded using NVivo (QSR International v11).

Intervention delivery fidelity was monitored by direct observation using two instruments (supplementary Appendix 2d). The Core Facilitator Behavioural Observation Sheet assessed 35 behaviours in six domains. Participant–educator interaction was assessed using the DESMOND Observation Tool.^24^ Every 10 s, the coder recorded whether an educator or participant was currently talking. Silence, laughter or multiple conversations were classed as ‘miscellaneous’. This provided an objective indication of facilitator versus participant talk time.

### Sample size

The sample size calculation was based on detecting a clinically meaningful difference of 4.5 kg (~5% reduction in body weight). This amount of weight loss is associated with improved lipid profile, glucose and blood pressure and potential reductions in cardiovascular disease.^25^ Based on previous UK data, we assumed a standard deviation (s.d.) of 10 kg. A total of 260 participants (130 per arm) were required to detect this weight difference assuming 95% power and a two-sided significance level of 5%. Based on an average of seven participants per group, and intraclass correlation of 5% in the intervention arm, the sample size was inflated by a design effect of 1.3 in the intervention arm yielding revised sample sizes of 169 and 130 in the intervention and control arms, respectively. To maintain a 1:1 allocation, 158 participants per arm were required. We anticipated a drop-out rate of 20% giving 198 participants per arm.

### Statistical analysis

All analyses were by intention to treat. The primary objective was assessed by fitting a marginal generalised estimating equation model adjusted for baseline weight, site and years since antipsychotic initiation; the model incorporated an adjustment for potential clustering or correlation among outcomes of people treated together. A sensitivity analysis assessed the robustness of the findings, in particular, to missing data mechanisms (including missing not at random), exploring whether the intervention had the same effect among recently diagnosed participants compared with those with longer illness duration. Other continuous outcomes were analysed and reported as for the primary outcome. Analyses were conducted using the Stata 14.2 software.

## Results

**Fig. 2:**
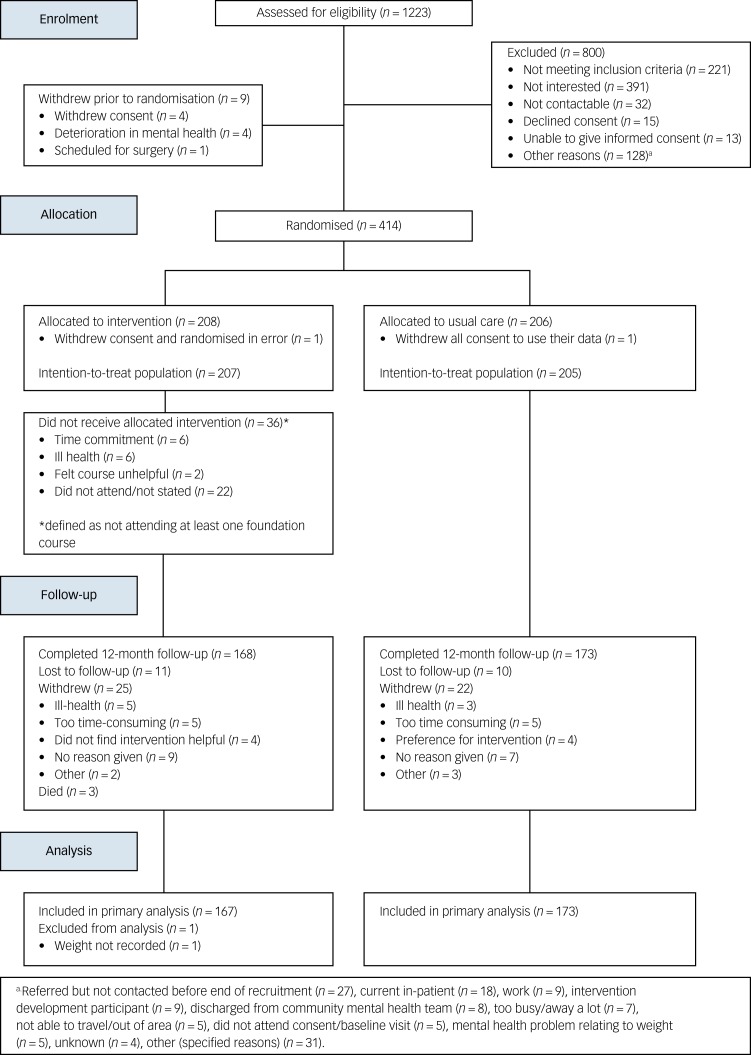
STEPWISE trial CONSORT diagram.

Between 10 March 2015 and 31 March 2016, we screened 1253 patients of whom 414 enrolled (Fig. 2). The trial closed on 31 March 2017 when the last 12-month follow-up was completed. The commonest reasons for exclusion at screening were ineligibility and lack of interest. Two participants withdrew consent prior to the study commencement and were not included in any analyses. Therefore, 412 participants (207 intervention, 205 control) were included in the final intention-to-treat analysis. In total, 168 (81.2%) intervention and 173 (84.4%) control participants completed the study, and 25 (12.1%) intervention and 22 (10.7%) control participants withdrew consent during the study. Eleven (5.3%) intervention and 10 (4.9%) control participants were lost to follow-up. Three deaths occurred in the intervention arm.

At baseline, the groups were largely balanced (Tables 1 and 2), but the intervention group were on average 3 kg heavier at baseline, partially explained by the higher proportion of men in the intervention arm (55.6% versus 46.3%). There were seven control and three intervention participants with a BMI <25 kg/m^2^. The OPCRIT+ concurred with the clinical diagnosis (supplementary Appendix 4.1). Participants reported mild-to-moderate psychiatric symptoms and took a range of antipsychotics (Table 1). Of those who completed the trial, 24 (14.3%) intervention participants and 29 (16.7%) control participants changed antipsychotic during the trial.

**Table 1:** Baseline characteristics

	Intervention (*n* = 207)	Control (*n* = 205)	Daily dose, mg: median (IQR)	
			Intervention (*n* = 207)	Control (*n* = 205)
Age, mean (s.d.)	40.0 (11.3)	40.1 (11.5)	–	–
Gender, *n* (%)				
Men	115 (55.6)	95 (46.3)	–	–
Women	92 (44.4)	110 (53.7)	–	–
Schizophrenia diagnosis type, *n* (%)				
ICD-10: F20	145 (70.0)	138 (67.3)	–	–
ICD-10: F25	30 (14.5)	36 (17.6)	–	–
First-episode psychosis	32 (15.5)	31 (15.1)	–	–
Time since starting antipsychotic medication, *n* (%)				
<1 year	12 (5.8)	12 (5.9)	–	–
1–2 years	11 (5.3)	20 (9.8)	–	–
2–5 years	28 (13.5)	19 (9.3)	–	–
5–10 years	28 (13.5)	33 (16.1)	–	–
10–20 years	71 (34.3)	69 (33.7)	–	–
20 or more years	57 (27.5)	52 (25.4)	–	–
Antipsychotic medication,^a^ *n* (%)				
Haloperidol (oral)	7 (3.4)	3 (1.5)	5 (5–10)	5 (1–9)
Amisulpride (oral)	21 (10.1)	16 (7.8)	400 (400–800)	175 (100–375)
Aripiprazole (oral)	37 (17.9)	28 (13.7)	10 (10–15)	10 (5–15)
Aripiprazole (long-acting injection)	3 (1.4)	6 (2.9)	14.3 (14.3–14.3)	14.3 (14.3–14.3)
Clozapine (oral)	89 (43.0)	81 (39.5)	300 (250–450)	350 (250–475)
Olanzapine (oral)	31 (15.0)	31 (15.1)	10 (5–15)	15 (10–20)
Quetiapine (oral)	28 (13.5)	24 (11.7)	350 (175–600)	250 (100–425)
Risperidone (oral)	6 (2.9)	16 (7.8)	4 (4–7)	4 (4–8)
Risperidone (long-acting injection)	4 (1.9)	5 (2.4)	3.1 (2.7–3.6)	2.7 (1.8–3.6)
Flupentixol (injection)	8 (3.9)	11 (5.4)	3.6 (3.2–5.0)	7.1 (2.9–14.3)
Zuclopenthixol (oral)	2 (1.0)	6 (2.9)	19 (10–28)	7 (6–20)
Zuclopenthixol (long-acting injection)	8 (3.9)	15 (7.3)	23.2 (11.9–32.1)	14.3 (12.1–35.7)
Paliperidone (long-acting injection)	7 (3.4)	8 (3.9)	5.4 (2.7–5.4)	3.6 (2.2–4.9)
Other antipsychotic	19 (9.2)	9 (4.4)	–	–
Ethnicity, *n* (%)				
White European	179 (86.5)	170 (82.9)	–	–
Asian	9 (4.3)	7 (3.4)	–	–
Black	12 (5.8)	19 (9.3)	–	–
Mixed	4 (1.9)	7 (3.4)	–	–
Other	3 (1.4)	2 (1.0)	–	–
Smoking status, *n* (%)				
Ex-smoker	55 (26.6)	52 (25.4)	–	–
Never smoked	54 (26.1)	45 (22.0)	–	–
Current smoker	98 (47.3)	108 (52.7)	–	–
Comorbid conditions, *n* (%)				
Abnormal renal function	60 (29.0)	58 (28.3)	–	–
Hepatic disease	5 (2.4)	7 (3.4)	–	–
Diabetes	35 (16.9)	25 (12.2)	–	–
Hypertension	21 (10.1)	17 (8.3)	–	–
Cardiovascular disease	7 (3.4)	12 (5.9)	–	–

IQR, interquartile range.

a. Where long-acting injectable medications have been used, the total dose has been divided by the dosing interval. Participants may have been taking more than one antipsychotic.

**Table 2:** Outcome measures at baseline, 3-month and 12-month follow-up visits^a^

	Baseline		3-month follow-up			12-month follow-up		
	Intervention group (*n* = 207)	Control group (*n* = 205)	Intervention group (*n* = 178)	Control group (*n* = 180)	Difference between intervention and control groups	Intervention group (*n* = 167)	Control group (*n* = 173)	Difference between intervention and control groups
Physical measures								
Weight, kg: mean (s.d.)	105.2 (22.2)	102.1 (22.1)	104.7 (21.5)	103.1 (23.5)	−0.55 (−1.44 to 0.35)	104.1 (21.1)	101.3 (23.7)	0.04 (−1.58 to 1.66)
% weight change, mean (s.d.)	–	–	−0.2 (4.4)	0.4 (4.7)	−0.4 (−1.3 to 0.5)	−0.5 (7.9)	−0.5 (8.3)	0.0 (−1.6 to 1.7)
Maintained or lost weight, *n* (%)	–	–	93 (52.2%)	80 (44.4%)	1.35 (0.88 to 2.05)^b^	98 (58.7)	88 (50.9)	1.35 (0.85 to 2.14)^b^
BMI,^c^ kg/m^2^: mean (s.d.)	36.1 (7.2)	35.3 (7.2)	35.8 (7.1)	35.5 (7.4)	−0.16 (−0.48 to 0.15)	35.6 (7.2)	34.8 (7.3)	0.05 (−0.51 to 0.61)
Waist circumference, cm: mean (s.d.)	117.8 (15.6)	116.1 (17.4)	116.8 (15.2)	115.4 (17.0)	0.79 (−0.64 to 2.22)	116.4 (16.1)	114.0 (17.7)	1.22 (−0.74 to 3.20)
Systolic blood pressure, mean (s.d.)	126 (16)	124 (17)	127 (16)	123 (16)	2.4 (0.2, 4.7)	125 (15)	122 (16)	1.7 (−1.1, 4.5)
Diastolic blood pressure, mean (s.d.)	82 (11)	82 (12)	82 (11)	81 (12)	0.4 (−1.5, 2.4)	82 (10)	81 (11)	1.1 (−0.7, 3.0)
Biochemical measures, mean (s.d.)								
HbA_1c_, mmol/mol	42 (13)	40 (11)	–	–	–	43 (15)	41 (14)	0.2 (−1.4 to 1.9)
Fasting glucose, mmol/L	5.9 (2.2)	5.8 (2.3)	–	–	–	6.4 (3.0)	6.0 (2.8)	0.2 (−0.2 to 0.7)
Total cholesterol, mmol/L	5.0 (1.2)	5.1 (1.2)	–	–	–	4.9 (1.2)	5.1 (1.1)	−0.2 (−0.4 to 0.1)
HDL cholesterol, mmol/L	1.2 (0.5)	1.2 (0.4)	–	–	–	1.2 (0.6)	1.2 (0.3)	0.0 (−0.1 to 0.1)
Triglycerides, mmol/L	2.5 (2.0)	2.2 (1.7)	–	–	–	2.4 (1.4)	2.4 (2.2)	−0.2 (−0.6 to 0.1)
Psychosocial measures, mean (s.d.)								
RAND (general health)	45.0 (20.3)	44.8 (20.7)	48.0 (21.8)	46.8 (20.3)	−0.3 (−3.4 to 2.8)	49.8 (23.1)	46.8 (21.4)	2.2 (−1.3 to 5.6)
EQ5D	0.793 (0.201)	0.783 (0.187)	0.815 (0.165)	0.785 (0.214)	0.02 (−0.02 to 0.054)	0.793 (0.237)	0.793 (0.239)	−0.02 (−0.06 to 0.03)
B-IPQ	5.5 (1.5)	5.5 (1.7)	5.0 (1.7)	5.3 (1.7)	−0.2 (−0.4, 0.0)	5.0 (1.9)	5.0 (1.7)	−0.0 (−0.3, 0.3)
BPRS	30.9 (8.8)	31.5 (9.4)	30.3 (9.0)	30.4 (9.4)	0.2 (−1.3, 1.7)	29.1 (9.7)	28.3 (9.5)	1.0 (−0.9, 2.9)
PHQ-9	10.6 (6.3)	11.0 (6.8)	10.3 (6.3)	10.1 (7.1)	0.5 (−0.4, 1.3)	9.9 (7.0)	9.6 (6.6)	0.5 (−0.4, 1.5)
Physical activity, mean (s.d.)								
MVPA^d^ (all days)	13.3 (16.8)	11.0 (13.1)	13.3 (20.4)	8.8 (12.6)	2.0 (−0.9 to 4.9)	15.4 (21.7)	11.8 (19.3)	1.5 (−2.5 to 5.5)
MVPA^d^ (weekends)	9.6 (16.6)	9.6 (14.8)	11.3 (24.9)	7.4 (12.4)	5.6 (2.0 to 9.3)	11.9 (22.1)	9.5 (19.2)	2.2 (−1.8 to 6.2)
MVPA^d^ (weekdays)	14.4 (18.5)	11.6 (14.8)	13.8 (20.3)	9.5 (14.3)	0.9 (−2.0 to 3.8)	16.6 (24.5)	12.6 (20.1)	1.0 (−3.9 to 6.0)
Mean acceleration (all days)	21.3 (7.9)	20.8 (7.4)	21.7 (9.0)	19.8 (7.1)	−0.4 (−1.5 to 0.8)	22.4 (8.2)	20.5 (8.5)	0.2 (−1.4 to 1.7)
Mean acceleration (weekends)	19.6 (8.0)	19.8 (8.3)	20.4 (9.6)	18.7 (6.9)	1.0 (−0.3 to 2.4)	20.9 (8.6)	19.4 (8.8)	0.3 (−1.5 to 2.1)
Mean acceleration (weekdays)	22.1 (8.3)	21.1 (7.1)	22.1 (9.2)	20.2 (7.5)	−0.7 (−2.0 to 0.6)	23.0 (8.5)	20.9 (8.6)	0.0 (−1.6 to 1.6)

B-IPQ, Brief Illness Perception Questionnaire; BPRS, Brief Psychiatric Rating Scale; PHQ-9: 9-item Patient Health Questionnaire.

a. Statistical analysis is on the basis of intention to treat.

b. Odds ratios with 95% confidence intervals.

c. Ten participants had a body mass index (BMI) below 25 kg/m^2^ at baseline (ranging from 19.5 to 24.9 kg/m^2^); none of these was from a South Asian or Chinese background.

d. Moderate-to-vigour physical activity (MVPA) is assessed in bouts >10 min in duration. Baseline accelerometery data were obtained from 85% of participants of whom 76% provided valid data (≥4/7 days). Comparative data were available for 54% and 52% of participants at 3 and 12 months.

### Intervention uptake

Participants commenced the STEPWISE intervention a median 15 days (range 1–101 days) after randomisation. Participants attended a mean of 2.7 foundation and 1.4 booster sessions. In total, 111 (53.6%) participants attended ≥3 foundation sessions and ≥1 booster session, of whom 47 (22.7%) attended all foundation and booster sessions. However, 36 (17.4%) participants attended no sessions. The mean group size at randomisation was 6.3 (median 6) but the mean number attending ranged from 4.0 to 4.4 (median 4) during the foundation course and dropped to 2.7–3.0 (median 3) during booster sessions (supplementary Appendix 4.2).

There were 169 (81.6%) participants who had one or more support contacts, mostly by telephone (80.7% participants, 2434 contacts), mail/postcard (49.3%, 555 contacts) or both (48.3%). Fewer participants were contacted electronically (11.6%, 88 contacts) or face to face (32.9%, 141 contacts). There were 25 (7.5%) participants (17 intervention and 8 control) who reported attending weight loss programmes outside the trial (supplementary Appendix 4.3).

### Outcome measures

The primary comparison of weight change at 12 months was almost identical between arms, with a mean reduction in weight of 0.47 kg in the intervention group and 0.51 kg in the control group (difference = 0.0 kg, 95% CI −1.6 to 1.7, *P* = 0.963) (Table 2 and supplementary Appendix Fig. 4.1). There was no difference in percentage weight loss or percentage of participants maintaining or losing weight.

Weight loss was modestly associated with age, with weight reduction increasing by 0.8 kg per 10 additional years (95% CI 0.0 to 1.5 kg, *P* = 0.042). Participants with schizoaffective disorder had greater mean weight loss (−2.7 kg) than those with first-episode psychosis (−0.3 kg) or schizophrenia (+0.01 kg; *P* = 0.023). There was no association between treatment effect and gender, baseline mental health, BMI, severity of psychiatric illness, duration and change of antipsychotic treatment, or attendance at an external weight loss programme. There was no association between total contact time and weight loss.

The baseline self-reported diet indicated a high consumption of refined sugar from sugary drinks and low fibre intake (supplementary Appendix 4.4). Although there was some evidence that alcohol intake fell in the intervention group, no other dietary component changed during the trial. Smoking status did not change (supplementary Appendix 4.5).

Both groups had similarly low physical activity levels at baseline (Table 2). After 3 months, weekend moderate-to-vigorous physical activity was significantly higher in the intervention group, but this difference had disappeared by 12 months. No other differences were seen in physical activity. Self-reported patient quality of life, obesity illness perception and psychiatric symptoms were also similar between groups at both 3 and 12 months (Table 2 and supplementary Appendix 4.6–4.7). The lack of objective changes in diet and lifestyle in the intervention group contrasted with self-reported changes during the ‘Sharing Stories’ part of the sessions.

At 3 months, outcome assessors were unmasked (or suspected unmasked) at 44 (12%) of visits (intervention: 34 of 178, 19%; control: 10 of 186, 5%). At 12 months, unmasking was recorded for 35 (10%) of visits (intervention: 31 of 168, 18%; control: 4 of 174, 2%).

The 703 anonymous participant session feedback forms showed 87.2% of respondents indicated the session met their needs (supplementary Appendix 4.8a). Forms were received from all ten sites with the number ranging 26 to 116 (supplementary Appendix 4.8b). Mean weight change did not correlate with mean centre feedback scores, at 3 or 12 months (Spearmans rho = −0.20, *P* = 0.476 and Spearmans rho = 0.042, *P* = 0.454, respectively).

Adverse events were similar between groups, except three deaths occurred in the intervention group; none were considered a result of the intervention (supplementary Appendix 4.9).

### Cost-effectiveness analysis

The two groups had similar EQ-5D-5L scores (Health Economics appendix). The intervention produced 0.0035 more QALYs. The mean total health and social care costs were £5255 for STEPWISE participants and £4453 for control participants. The mean total societal costs were £11 332 for STEPWISE participants and £10 305 for control participants. The incremental cost-effectiveness ratio from the healthcare perspective is £246 921 and £367 543 from the societal perspective.

### Process evaluation

Facilitator and participant courses were popular, and materials were adequately resourced, although doubts were expressed about financial sustainability. Professionals were generally motivated but expressed the concern that in some trusts human resource and leadership support were inadequate.

Fidelity assessment of intervention delivery showed overall mean  percentage facilitator talk time was 47.6% (s.d. = 12.3%) (supplementary Appendix 4.10). ‘Positive’ (more facilitative) behaviours were observed for 54.1% (s.d. = 15.0%) of the time. Conversely, ‘negative’ (more didactic) behaviours were observed for 23.8% (s.d. = 15.4%) of the time. Problems with fidelity included facilitators giving insufficient time for answering questions or completing tasks as well as providing rather than eliciting solutions from participants. Although the session structure provided dedicated space for participants to share their behavioural change successes and challenges, the intervention incorporated no objective assessment of whether participants had understood and were acting on programme content. There was difficulty delivering telephone support contacts, commonly because participants did not answer.

## Discussion

### Main findings

The STEPWISE trial successfully recruited and retained participants; however, the intervention was neither clinically nor cost-effective over the 12-month intervention period. Both groups lost ~0.5 kg but weight change did not differ between groups. There was no sustained behaviour change in diet and physical activity needed to promote weight loss.

These results were unexpected as previous studies had indicated that non-pharmacological interventions could support weight reduction;^3^ however, most studies had fewer than 100 participants, were of short duration, at moderate risk of bias and demonstrated substantial heterogeneity of effect size.^11^ NICE concluded that lifestyle interventions could reduce body weight in the short term but effects beyond 6 months were unknown.^11^

Given our findings, we examined why the intervention did not work and the implications for future research and clinical practice. In terms of trial conduct, recruitment exceeded our target and satisfactory retention and data completeness for the primary outcome ensured the trial was adequately powered. The 1-year follow-up allowed a long-term perspective and assessor masking reduced the risk of bias.

STEPWISE was robustly developed in collaboration with people with schizophrenia and met UK Department of Health guidelines for structured education.^26^ The intervention was pragmatic, theory-based, feasible and appeared acceptable to both people with schizophrenia and mental healthcare professionals.^27^ Direct observation of sessions, the gold-standard method for investigating fidelity, demonstrated that, despite the higher than expected turnover of facilitators, the intervention was delivered as planned and tailored appropriately.

### Comparison with findings from other studies

Although our findings are at odds with the effects of short-term interventions, other long-term studies have failed to demonstrate a benefit of lifestyle intervention. A recent meta-analysis found significant weight loss in only two of six studies with interventions lasting longer than a year.^7^ The Danish CHANGE study, which randomised 428 people with schizophrenia spectrum disorders and abdominal obesity to 12 months of intensive lifestyle coaching plus care coordination plus usual care, or care coordination and usual care, or usual care alone, found no effect on body weight or waist circumference with either intervention.^6^ Two other recent UK lifestyle intervention trials have also not met their primary outcome.^2^,^8^^,^^2^,^9^

It is instructive to compare the results of STEPWISE and CHANGE with two large US trials where weight loss was achieved. In the ACHIEVE study, the intervention group lost on average of 3.2 kg over 18 months^4^ whereas in STRIDE, intervention participants lost 4.4 kg more than control participants from baseline to 6 months but this difference reduced to 2.6 kg at 1 year.^5^ Both the ACHIEVE and STRIDE interventions were considerably more intensive than STEPWISE. ACHIEVE combined group weight-management sessions (weekly in the first 6 months then monthly), monthly individual visits and thrice weekly group activity classes, whereas the STRIDE study involved a 6-month weekly group intervention followed by a total of six monthly maintenance sessions.

The maximum face-to-face contact time in STEPWISE (17.5 h) is similar to that recommended by the NHS Diabetes Prevention Programme and it is debatable whether a more intensive intervention would be feasible within many healthcare settings. Even accounting for the lower cost of delivering STEPWISE in real-world clinical practice, a more intensive programme would likely be unaffordable, a concern raised by several facilitators. In STEPWISE, despite the successful pilot study and use of motivational techniques to engage participants, intervention uptake was challenging, as judged by the number of sessions attended, although the level of engagement was similar to other group-based education programmes.^30^^,^^3^,^1^

Intervention intensity, however, does not fully explain why STEPWISE was unsuccessful as the unsuccessful CHANGE study included weekly 1-hour sessions for a year. Both STEPWISE and CHANGE recruited people with schizophrenia; by contrast, 41.9% of ACHIEVE participants and 71% of STRIDE participants had mental illness other than schizophrenia spectrum disorders, for whom behaviour change may be easier to achieve. Whether STEPWISE would have been more successful for those with other psychotic illnesses, such as bipolar disorder, is unknown.

### Strengths and limitations

By design, we included a broad representation of people with schizophrenia and first-episode psychosis, although those with high levels of psychiatric symptoms were excluded. The participants had a spectrum of BMI from normal weight to morbid obesity. Most had a long history of established psychiatric disorder and around 40% were taking the second-line antipsychotic, clozapine. It is possible that the intervention could have been more effective during early psychosis, when weight gain is most rapid.^2^ Although we planned to include individuals shortly after the diagnosis of first-episode psychosis, few participants had received treatment for less than 3 months, partly because of delays inherent in recruiting to a group intervention.

To achieve meaningful weight loss, sustained behaviour change is needed. At baseline, participants ate an unhealthy diet and were physically inactive. Despite an opportunity to make a change, the intervention had little impact. One limitation of the intervention was the lack of objective feedback about participants’ progress to facilitators. The process evaluation indicated that facilitators wanted more information about participant weight change and nutritional and exercise plans to check understanding of session content and monitor dietary or physical activity changes against action plans.

Notwithstanding the negative results, the trial has important findings. Despite concerns about undertaking trials in this population, we successfully delivered the largest trial in this area with a 12-month follow-up across a diverse group of community mental health teams. We achieved our recruitment target 3 months ahead of schedule and maintained participants throughout the year-long trial. The trial also highlighted patient and healthcare professional demand for weight-management programmes within mental health settings and, in response, several trusts increased their physical health monitoring and engagement with weight management. Participants also valued sharing experiences with other people with schizophrenia with similar weight problems.

### Implications

The challenge of managing obesity and weight gain in people with schizophrenia remains and other approaches are needed. STEPWISE focused on lifestyle modification rather than the breadth of contributors to weight gain and obesity. Antipsychotics are associated with weight gain while psychosis and psychological factors can impede weight loss behaviours. Broader approaches that combine individually tailored lifestyle modification with psychological interventions for mental health, adjustment of antipsychotic treatment or co-prescription with drugs, such as metformin, may be needed.^32^

Although it is clear that lifestyle change is needed for people with schizophrenia, STEPWISE has shown how difficult this is to achieve. NICE guidance currently recommends ‘people with psychosis or schizophrenia, especially those taking antipsychotics, should be offered a combined healthy eating and physical activity programme by their mental healthcare provider’.^11^ Before these lifestyle interventions are commissioned across the NHS, it is vital that further research is undertaken to address how best to support weight management.

## Appendix

### The STEPWISE Research Group

University of Southampton: Richard I. G. Holt (chief investigator), Katharine Barnard. University of Sheffield: Rebecca Gossage-Worrall (research associate), Mike Bradburn (senior statistician), Daniel Hind (CTRU assistant director), David Saxon (statistician), Lizzie Swaby (research assistant). Greater Manchester Mental Health NHS Foundation Trust: Paul French (principal investigator), John Pendlebury (community psychiatric nurse – retired). Leeds and York Partnership Trust: Stephen Wright (principal investigator). Sheffield Health and Social Care NHS Foundation Trust: Glenn Waller (principal investigator). Kings College London: Paul McCrone (health economist), Tiyi Morris (research assistant). University of Leicester: Charlotte Edwardson (associate professor in physical activity, sedentary behaviour and health), Kamlesh Khunti (professor of primary care diabetes and vascular medicine), Melanie Davies (professor of diabetes medicine). University Hospitals of Leicester: Marian Carey (director: structured education research portfolio), Yvonne Doherty (consultant clinical psychologist), Alison Northern (project manager), Janette Barnett (diabetes specialist nurse). Cornwall NHS Trust: Richard Laugharne (principal investigator). Devon Partnership Trust: Chris Dickens (principal investigator). Somerset Partnership Trust: Chris Dickens (principal investigator). Sussex Partnership: Kathryn Greenwood (principal investigator). South London and Maudsley NHS Foundation Trust: Fiona Gaughran (co-principal investigator), Sridevi Kalidindi (co-principal investigator). Southern Health NHS Foundation Trust: Shanaya Rathod (principal investigator). Bradford District Care Trust: Najma Siddiqi (principal investigator). Angela Etherington (independent service user consultant), David Shiers (carer collaborator).

## Supplementary material

For supplementary material accompanying this paper visit https://doi.org/10.1192/bjp.2018.167. click here to view supplementary material
