# Supplementary material for: Structured lifestyle education for people with schizophrenia, schizoaffective disorder and first-episode psychosis (STEPWISE): randomised controlled trial
Source: Br J Psychiatry. 2019 Feb;214(2):63–73. doi: 10.1192/bjp.2018.167 (PMC6330076; doi:10.1192/bjp.2018.167)
Supplement: Supplementary file 1 [file S0007125018001678sup001.docx]

**Appendix 1: The STEPWISE intervention**

**Development of the STEPWISE intervention**

The intervention was developed using the internationally recognised MRC framework for complex interventions. Many weight loss programmes involve one-to-one strategies to promote behaviour change but these are unlikely to be affordable in many healthcare settings. Group-based structured education offers an alternative cost-effective delivery approach to promote self-management and behaviour change (1), and has been adopted by the UK NHS Diabetes Prevention Programme (2). Synonyms for structured education include self-management education programme.

The NICE approved Diabetes Education and Self-Management for Ongoing and Diagnosed (DESMOND) programme has robust pathway and framework to develop structured education programmes for people with diabetes and at risk of diabetes. DESMOND has been exported outside the UK and has trained thousands of people with diabetes in Australia. DESMOND is also being delivered successfully in Qatar and is being trialled in Mozambique and Malawi having been successfully piloted with local populations there. DESMOND programmes have been developed for people from ethnic minorities and people with learning difficulties.

The key criteria for a structured education programme are:

- A clear underlying philosophy which is grounded on established psychological theories of behaviour change
- A structured written curriculum
- Trained educators who are familiar with the programme and its delivery
- A quality assurance system to cover the structure, process, content, and delivery of the programme
- An audit process to monitor biomedical and psychosocial outcomes as well as patient experience

As the quality assurance and audit systems are only introduced once the programme is implemented, only the first three criteria were applied when developing the STEPWISE intervention.

We used the established DESMOND pathway and framework and embedded the core philosophies of DESMOND into the STEPWISE intervention.

**Literature review**

We first performed a literature review across the PsycInfo, Medline, PubMed, CINAHL, and Cochrane Library databases, using the search terms, ‘weight,’ ‘antipsychotic,’ and ‘intervention’ plus ‘behavioral,’ ‘psychoeducation,’ exercise,’ or ‘cognitive’. These terms had been used in an earlier meta-analysis of non-pharmacological interventions by Caemmerer and colleagues (3). This reported the findings of 17 studies and concluded that behavioural interventions could be beneficial in significantly attenuating weight gain and lowering body mass index (BMI) in people receiving antipsychotic medication, at least in the short term. Our updated literature review identified one additional study.

The duration of the interventions ranged from 12 to 24 weekly sessions, and the interventions were delivered in both groups and individually. Most of these studies were conducted in single centres and none were conducted in the UK. The content of the intervention included the setting of a variety of nutritional and physical activity targets. No differences in effect were found between modalities, duration and group versus individual delivery. The only difference identified was that out-patient interventions appeared more effective than in-patient interventions, although there was some evidence to suggest that nutritional interventions may have a greater effect than cognitive behavioural therapy. A theoretical basis was only reported for one study that specifically employed social cognition theory.

**Stakeholder meetings**

The STEPWISE intervention development involved a collaboration between a team with expertise in the development of obesity and lifestyle intervention programmes, mental healthcare professionals, researchers with specialist knowledge of the needs of people with schizophrenia and psychosis and the input of service users and participants.

It became apparent from the initial meetings with stakeholders, the literature review, and expert opinions of clinicians and practitioners actively providing local weight management interventions for people with psychosis, that although the guiding principles underpinning development of the DESMOND programme remained relevant, none of the existing DESMOND programmes was suitable for STEPWISE target participants.

We recognised that the length of sessions needed to take account of the concentration difficulties experience by some people with schizophrenia. The importance of long term follow-up and support was appreciated.

**Theoretical Framework**

We considered three key areas that are core to weight management interventions in people with schizophrenia which guided the overall intervention (appendix figure 1.1):

- behaviour change theory specifically with a focus on food and physical activity;
- psychological processes underlying weight management;
- the challenges of living with psychosis and its impact on eating and weight.

Based on a number of psychological theories, appropriate behaviour change techniques were used to address key hypothesised problem behaviours (appendix table 1.1).


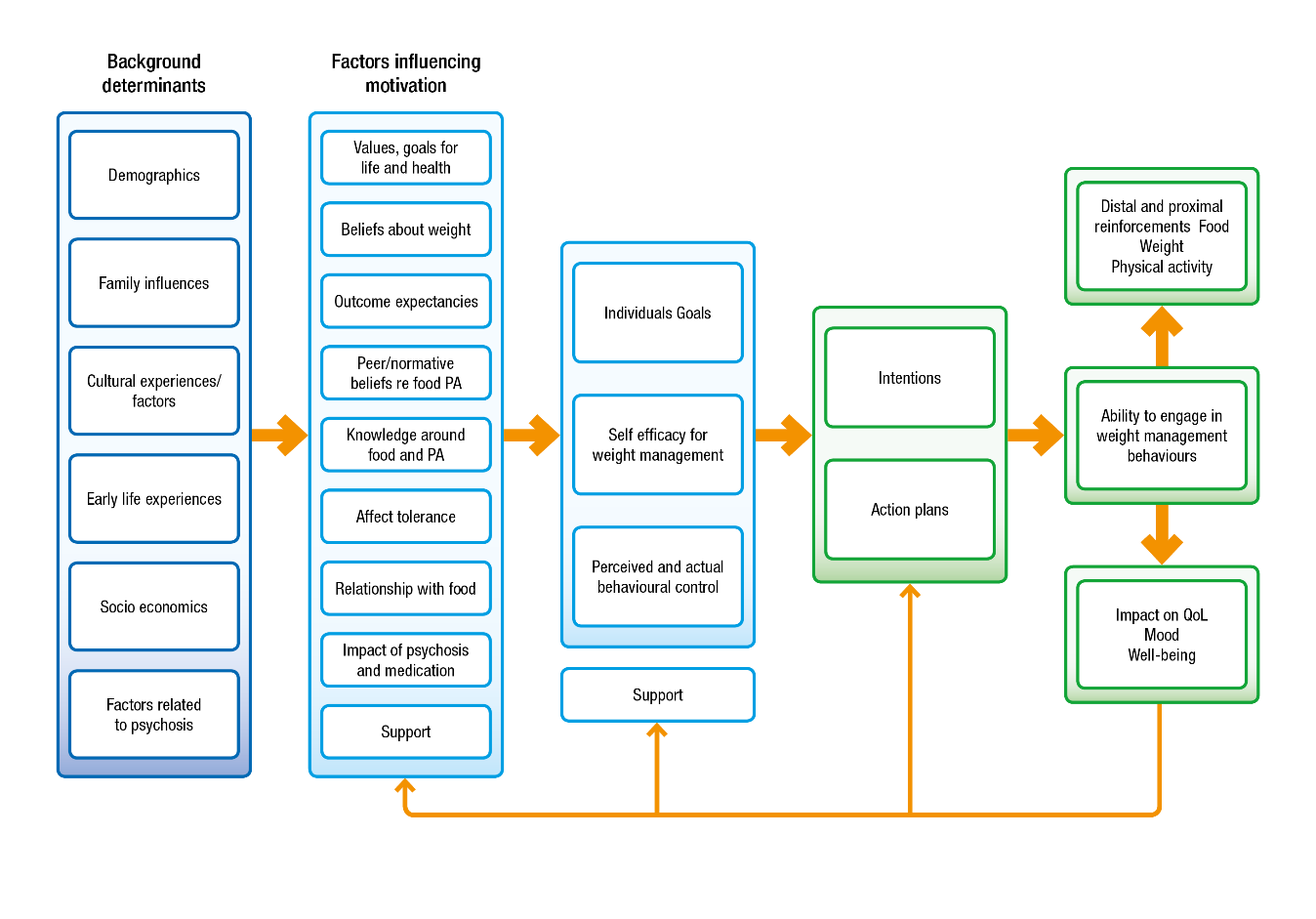


**Appendix figure 1.1: Theoretical Framework of the STEPWISE intervention**

**Appendix Table 1.1: Development of the Intervention based on the three core theories**

| Identified target behaviour/problem | Theory | Participant receipt and potential behavioural outcome | Intervention on the STEPWISE Course | Mapping to behavioural taxonomy |
| --- | --- | --- | --- | --- |
| Beliefs about weight problems, e.g. it is entirely due to their medication and therefore they can have no impact on their weight | Self-regulation Theory  Specifically illness representations around weight management   - Signs of a weight problem - Causes - Consequences - Treatment - Controllability - How long it will last | To have identified their own potential erroneous beliefs and challenged these in order to directly influence their decisions and strategies around weight management. | **Your story Session**  In session 1: Elicit participants’ beliefs about what caused their weight problem, what ‘treatment’ would help to manage it, the consequences for them and their health.  **Topic Sessions**  Information sessions throughout the course. Specifically the impact of medication on their weight, and the strategies they can employ to manage their weight | Not completely specified but included in:   - Information about health consequences - Framing/reframing |
| Low levels of confidence around being able to engage in successful weight management possibly related to multiple unsuccessful attempts at sustained weight loss. | Self-Efficacy   - Mastery (previous successful attempts of the behaviour) - Modelling (observing others engaging in the behaviour) - Verbal Persuasion (talking through the process of change expecting success) - Emotional arousal (managing the emotional barriers to change particularly anxiety around change and fear of failure) | Increased belief in their ability to engage successfully in weight management.  Identified strategies to increase their self-efficacy, including barriers in their environment and engage in behaviour change | **Sharing Stories Session**  At the start of each session: Elicit and record what has gone well in terms of specific behaviour changes made, problem solving around challenges and barriers to the action plans they set and observing and learning from others’ successes and shared problem solving.  Discuss feelings as activators and barriers to change, including low self-esteem.  **Next STEPS**  At the end of each session: Action planning, identifying barriers problem solving setting small graded tasks | - Focus on past successes - Self-monitoring of behaviour outcomes of behaviour and consequences - Instruction on how to perform the behaviour - Graded tasks - Behavioural experiments - Credible source - Habit reversal - Review behavioural goals - Social comparison - Focus - Goal setting - Action planning - Problem solving - Information about antecedents - Information about emotional consequences - Reduce negative emotion - Self-incentive - Self-reward |
| Maintenance of behaviour change particularly as there are strong cues to previous behaviours and thus high likelihood of relapse | Relapse Prevention Model   - High-risk situations with strong cues need to be managed by avoidance or coping strategies. - Coping strategies need to be prepared in advance - Management of relapse will result in increased self-efficacy | Reviewed the situations that would most likely result in relapse. Developed plans of how to manage these when they occur.  View relapse as a natural part of the change process and as an opportunity to learn rather than berate themselves and reinforce a potential negative self-perception or low self-esteem. | **Keeping it Going**  Visual tools and interactive exercises to explore potential sources of relapse and develop plans to overcome these when they occur. | - Self-monitoring of behaviour - Information about antecedents - Behaviour assessment - Goal setting - Problem solving - Action planning - Review behavioural goals - Restructuring physical and social environment - Avoidance/reducing exposure to cues for behaviour - Reduce negative emotion - Prompts - Remove access to the reward - Framing/reframing - Verbal persuasion about capacity |

**The development of the STEPWISE intervention**

The process and behaviour change techniques were informed by the literature review, stakeholder meetings and theoretical framework described above. The style of the session was based on an adult-based constructivist approach, encouraging participants to link learning to their own experience. The aim of the intervention was to help individuals develop their self-efficacy rather than change their social milieu. The intervention used behaviour change techniques, based on existing evidence-based psychological theories to support people with schizophrenia, schizoaffective disorder and first episode psychosis to achieve weight management, including weight loss.

The curriculum content was determined by the unique challenges that people with schizophrenia face in relation to food and physical activity. We recognised that antipsychotic medication affects thirst and the experience of satiety and thus people report drinking larger quantities of sugary drinks and have difficulties with portion control. Low vitality reduce physical activity levels and the ability to engage in cooking.

In line with existing psychological theories, including the benefits of self-monitoring, operant conditioning and underpinned by an empowerment philosophy, participants were provided with tools to support desired behaviours. These included a water bottle (Healthier drinks), a pedometer (Physical activity), cookery books and kitchen scales (Calories and portions) and weighing scales and tape measure (Taking control of your weight).

When designing the intervention we had to balance the benefits of increased contact time with the need to develop an intervention that would be affordable and implementable within the constraints of the NHS. The prototype intervention was designed to have 17.5 hours face-to-face group contact time plus a further 10 minutes support contact predominantly by phone, every fortnight (total ~8 hours). The level of face-to-face contact time is similar to the UK NHS diabetes prevention programmes, which was recommended following a review of the literature about the minimal contact time needed to support behaviour change.

**Piloting the STEPWISE intervention**

The four foundation intervention sessions were piloted within Sheffield Health and Social Care NHS Foundation Trust in 20 people with schizophrenia in four cycles between May and December 2014 to test the acceptability and feasibility of content and delivery. The inclusion and exclusion criteria were the same as the main trial. After each cycle, the intervention was amended following feedback from participants and facilitators.

During the pilot phase, a user-led, local mental health support group in Leicester comprising people with schizophrenia and other mental health conditions such as depression and bipolar disorder, provided further advice about the curriculum, resources and delivery logistics. The mental health support group discussed challenges raised during the pilot with the research team such as recruitment and retention.

The pilot study showed that the intervention was acceptable and engaged people with schizophrenia. Overall, feedback from both the participants and facilitators was positive, with self-reported behaviour change by most individuals. The participants highlighted the importance of a safe and non-judgmental environment in a small familiar setting. The participants either endorsed aspects of the intervention or recommended a number of modifications to improve recruitment and retention in the main trial:

1. Logistics
   1. The provision of transportation was noted as fundamental to enabling participants to attend on time and maintain attendance. In response, we organised taxis to bring the participants from home to the venue.
   2. Time of the sessions. As people with schizophrenia often have altered sleep patterns, the sessions were planned at lunch time. We asked people to arrive at 1230 in order to be ready for a 1300 start. A healthy lunch was provided on arrival, which had two benefits. First, sessions were not disrupted if participants arrived late and, second, we were able to provide a practical demonstration of ways to eat healthily on a limited budget.
   3. Overall the participants commented that the duration of each session (2.5 hours) was appropriate.
2. Concentration
   1. In recognition of many participants’ impaired ability to concentrate, we incorporated 1-2 breaks into the session and trained facilitators to introduce these at suitable points in the session, whilst maintaining the momentum of the delivery.
3. Session delivery
   1. Participants and facilitators described the use of resources, such as flipcharts, laminates, booklets and resources as valuable and engaging.
   2. Participants reported the benefits of using the same facilitators and participants throughout the intervention because they became “part of the group”.
4. Incentives and motivation
   1. The introduction of supporting tools reinforced the key messages of the intervention and improved internal motivation while supporting engagement and attendance.
5. Accompanying person
   1. Contrary to our expectation, the participants expressed a strong view that they did not wish accompanying persons, such as family members or carers, to attend as participants wanted to accomplish attendance at the sessions on their own.

A full description of the development of the STEPWISE intervention is currently in submission to *Pilot and Feasibility Studies*.

**The STEPWISE intervention**

The final intervention comprised four 2.5 hour foundation group education sessions, designed to be delivered to small groups of 6-8 participants over four consecutive weeks followed by three 2.5 hour follow-up ‘booster’ sessions which took place at months 4, 7 and 10 after randomisation, and starting approximately 3 months after the end of the foundation sessions (figure 1, main paper). In addition, fortnightly support, usually by telephone, was planned to reinforce the learning from the sessions and support action plans. A written curriculum was prepared to direct intervention delivery to ensure consistency.

All sessions were arranged to start at lunchtime with the provision of a healthy lunch. Flexible breaks were incorporated into the intervention to take account of participants’ specific issues with concentration (figure 1.2).

Figure 1.2: STEPWISE intervention


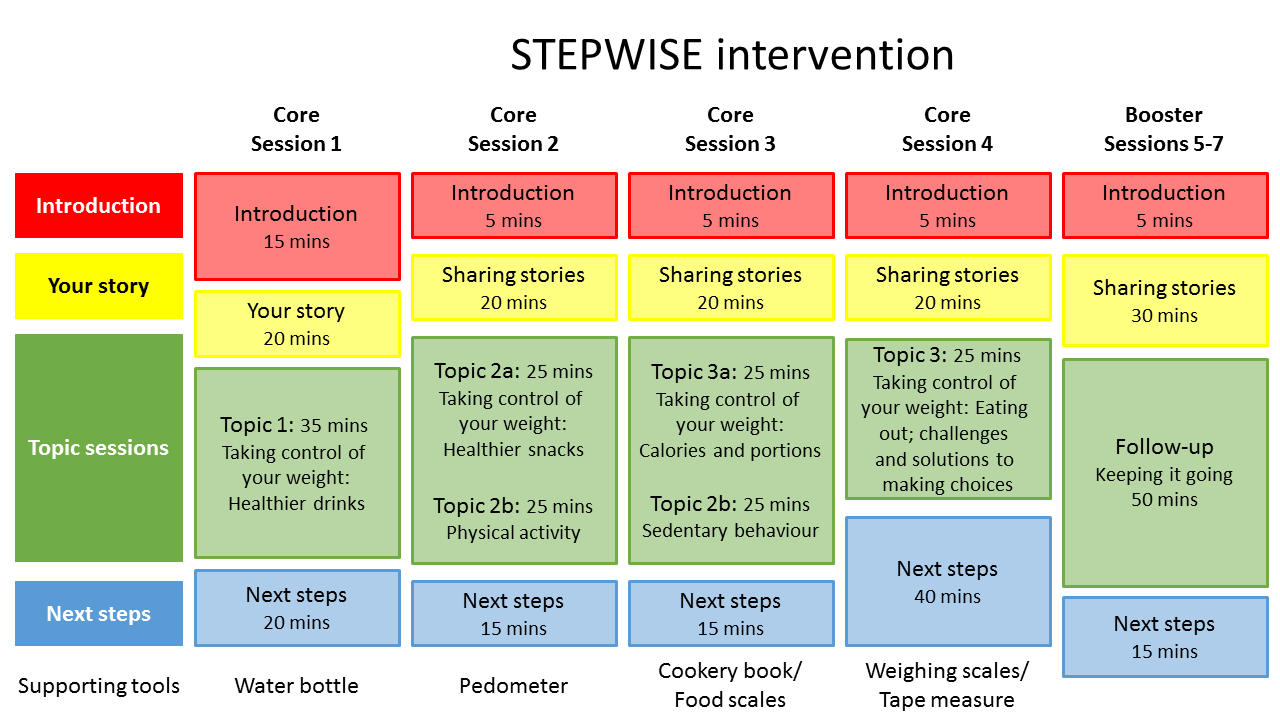


After an initial introduction, all sessions started with participants being invited to “share their story”. This process entailed participants describing what they had achieved thus far specifically in relation to the goals and action plans they had developed in the proceeding session. This provided the facilitators with feedback on what changes the person had been able to make and what had proved challenging. As with the rest of the programme a non-judgemental style was employed to encourage openness about individuals’ challenges and encourage problem solving and sharing successful strategies within the group. Specific changes and challenges were recorded on flipcharts and discussed. Examples of self-reported behaviour changes included:

- Various changes in food choices:
  - Reduction in the consumption of sugary carbonated drinks by changing to diluted orange juice or water.
  - Another participant recommended having a bottle of water at hand in case of feeling thirsty.
  - Others reported that when they felt well, they would cook extra portions of healthy meals that could be frozen in readiness for ‘bad’ days.
  - Reductions in eating fast food or take-away meals
- Physical Activity
  - One participant reported taking up cycling again on a regular basis, an activity he had previously allowed to lapse.

All the participants’ strategies for successful behaviour change were noted and collected across all the sessions so that the participants could refer back to their own individualised solutions. The types of problems and solutions shared were captured in a series of qualitative interviews with participants and educators, and in observation notes from the formal intervention fidelity visits. The flipcharts were not kept after the final session and their content was not formally analysed.

Although the participants were not weighed at the start of the sessions at their request, they were encouraged to use the provided weighing scales to weigh themselves in private and discuss their progress in the groups if they felt comfortable in doing so.

The next part of the session was entitled ‘Taking control of your weight’ in order to reinforce the focus of the intervention. Each session covered one or two aspects of how lifestyle changes could help the participants take control of their weight during the intervention. Four topics covered diet while two focussed on physical activity. Sessions also included the impact of medication on thirst and satiety and the strategies that might help people to overcome these challenges. The specific topics included:

- Cutting calories by choosing lower calorie drinks (Healthier drinks)
- Cutting calories by choosing lower calorie snacks (Healthier snacks)
- Cutting calories by reducing portion size, eating slowly and focusing on what participants were eating and using other cues as a trigger to stop, rather than feeling full (Calories and portions)
- Cooking on a budget, reducing calories from takeaway food and ready meals (Eating out; challenges and solutions to making choices)
- The importance of physical activity was emphasised by using Thera-Bands, with free Thera-Bands being made available to participants. In addition, all participants were given a pedometer and encouraged to monitor their step count (Physical activity)
- The importance of reducing sedentary time (Sedentary behaviour)

During these sessions, the facilitators were encouraged to adopt a facilitative approach as opposed to teaching in a didactic manner to enable the participants to discuss their beliefs about weight and explore own solutions. Discussions also included the effects of medication on weight and physical activity.

The final section of the sessions was devoted to action planning (Next Steps) during which time the participants were encouraged, with the support of the facilitators, to develop their own individualised goals and to consider how they could incorporate the learning into their everyday lives.

Appendix table 1.2 gives a breakdown of the time devoted to different activities in the foundation and booster sessions.

| **Activity** | **Foundation Sessions** | **Booster Sessions** |
| --- | --- | --- |
| Introduction | 8% | 9% |
| Beliefs about weight management | 5.5% |  |
| Sharing success and problem solving | 17% | 27% |
| Medication and weight management | 5.5% |  |
| Personalised changes to food and drinks | 19% |  |
| Personalised changes to eating habits | 10% |  |
| Mindfulness and food |  | 8% |
| Managing food changes on a budget |  | 7.5% |
| Personalised changes to activity and sedentary time | 14% | 7.5% |
| Goal setting and action planning | 21% | 18% |
| Relapse prevention strategies |  | 23% |

At the end of each session, before the participants departed, they were given supporting tools to reinforce the key messages that had been discussed during the session. In addition to free samples of low calorie drinks and snacks, these included a water bottle (Healthier drinks), a pedometer (Physical activity), cookery books and kitchen scales (Calories and portions) and weighing scales and tape measure (Taking control of your weight). The cookery books used every day accessible and affordable ingredients. By offering the tools, we aimed to support the participants in achieving their action plan goals and maintain their attendance the later sessions.

The foundation sessions were enhanced by three 2.5 hour follow-up ‘booster’ sessions which took place at months 4, 7 and 10 after randomisation, and starting approximately 3 months after the end of the foundation sessions. These were designed to reinforce the original messages and support continued behaviour change. Fortnightly support, usually by telephone, was planned to reinforce the learning from the sessions and support action plans.

**STEPWISE intervention delivery**

The intervention was designed to be delivered by two local specifically trained facilitators, who worked in mental health. At least one of the facilitators was a registered mental health professional while the other was required to have a professional background as either a registered mental health professional, mental health support worker, healthcare assistant or similar. Current experience of working with people with mental health issues and knowledge of antipsychotic medication were essential facilitator attributes.

58 facilitators from the 10 participating organisations were trained and delivered the intervention. 42 were trained to deliver the whole intervention (foundation and booster sessions and support contacts). Six attended training for the booster sessions only, eight attended all training except the booster session training and two attended all training except the support call session training.

The high number of facilitators reflects the reality of frequent movement of staff within mental health services in the UK. Two organisations were also affected by major service configuration during the study (merging of mental health trusts), which added to the pressures on delivery. Although it is preferable in interventions of this kind to have a continuity of facilitators, for pragmatic reasons, such as staff availability, emergency situations, holiday and sickness absence, maternity leave etc., often continuity could only be achieved for one facilitator.

In a ‘real world’ setting as opposed to a research environment, interventions such as STEPWISE would be embedded in the pathway of care, and ongoing support for whatever goals participants set for themselves, would be provided by key workers and other service personnel to sustain motivation and progress.

References

1. NICE. National Institute for, Health Clinical, Excellence. Preventing type 2 diabetes: risk identification and interventions for individuals at high risk. PHG38. 2012.

2. NHS England. NHS Diabetes Prevention Programme (NHS DPP). <https://www.england.nhs.uk/diabetes/diabetes-prevention/>.

3. Caemmerer J, Correll CU, Maayan L. Acute and maintenance effects of non-pharmacologic interventions for antipsychotic associated weight gain and metabolic abnormalities: a meta-analytic comparison of randomized controlled trials. Schizophr Res. 2012; 140(1-3): 159-68.

**Appendix 2: Methodology**

**Appendix 2a: Anonymous session feedback form**

**Appendix 2b: Standard operating procedures for taking body measurements and accelerometry data**

A trial-specific standard operating procedure for taking the body measurements, required by the protocol, was written by the Trial Manager (RGW) and approved by the Chief Investigator (RIGH). We include a summary of the instructions provided to all sites during set-up. The equipment manuals were referenced in the standard operating procedure and provided along with a carry case (where available). Research staff were trained to document any use of alternative measuring equipment to that provided by the Clinical Trials Research Unit; and, asked to state any alternative (equivalent class) equipment used with reasons on the Case Report Form.

## Weight

Research staff were provided with portable Class III approved Marsden 420 C weighing scales. The standard operating procedure specified that: a) scales be placed on an uncarpeted, level floor and zeroed before use; and, b) weight be measured in kilograms (kg) to one decimal place. The participant was asked to remove outdoor clothing (e.g. shoes) and heavy items from pockets.

## Height

Research staff were provided with a portable Class 1 approved Marsden HM-250P stadiometer to measure height to the nearest centimetre. Participants were asked to remove their shoes and anything else that might restrict or impact on measurement (e.g. hair dressing), stand straight and tall against the perpendicular post with arms loosely by their side and head level.

## Waist circumference

Research staff were provided with a Class 1 approved Seca 201 tape to measure waist circumference in centimetres (cm) to the nearest 0.1cm. The standard operating procedure specified: a) where to locate the participant’s waist and correct positioning of the tape (e.g. flat and level; taut but not tight); and, b) that measurement was taken with the participant wearing light clothing, arms by their side and standing with feet approximately 25-30 cm apart.

## Blood pressure

The protocol specified that blood pressure (BP) be taken using electronic sphygmomanometer (not provided by the study) in the non-dominant arm after 5 minutes rest, record three measurements in millimetres of mercury (mmHg) and document an average on the CRF.

**Appendix 2c: GENEActiv accelerometry**

Participants were asked to wear the wrist worn GENEActiv (Activinsights Ltd, Kimbolton, UK) accelerometer on their non-dominant wrist continuously (i.e. 24 h/day) for 7 days to assess physical activity. GENEActiv .bin files were analysed with R-package GGIR (<http://cran.r-project.org>) (van Hees et al 2013; van Hees et al 2014). Signal processing in GGIR includes the following steps:

1. autocalibration using local gravity as a reference (van Hees et al 2014)
2. detection of sustained abnormally high values
3. detection of non-wear calculation of the average magnitude of dynamic acceleration (i.e. the vector magnitude of acceleration corrected for gravity (Euclidean Norm minus 1 *g*) as over 5 s epochs with negative values rounded up to zero.

*
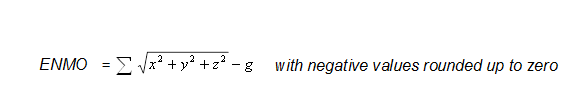
*

Files were excluded from all analyses if post-calibration error was greater than 0.02 *g* (da Silva et al 2014) or fewer than 16 h of wear-time were recorded by either monitor during the 24 h day of interest. Detection of non-wear has been described in detail previously (See ‘Procedure for non-wear detection’ in supplementary document to van Hees et al 2013). In brief, non-wear is estimated based on the standard deviation and value range of each axis, calculated for 60 min windows with 15-min moving increments. If for at least 2 out of the 3 axes the SD is less than 13 m*g* or the value range is less than 50 m*g* (milligravity) the time window is classified as non-wear.

The average magnitude of dynamic wrist acceleration (ENMO) and time accumulated in moderate-to-vigorous physical activity (MVPA) were calculated. The threshold for determining MVPA was ≥100 m*g* (Hildebrand et al 2014). MVPA duration was calculated as sustained MVPA (i.e., all bouts performed for at least 10 minutes duration with 80% of epochs within this threshold).

References:

van Hees VT, Fang Z, Langford J, Assah F, Mohammad A, da Silva IC, et al. Autocalibration of accelerometer data for free-living physical activity assessment using local gravity and temperature: an evaluation on four continents. J Appl Physiol.2014;117(7):738-44.

Van Hees VT, Gorzelniak L, Leon ECD, Eder M, Pias M, Taherian S, et al. Separating movement and gravity components in an acceleration signal and implications for the assessment of human daily physical activity.PLoS ONE. 2013;8(4):e61691.

da Silva IC, van Hees VT, Ramires VV, Knuth AG, Bielemann RM, Ekelund U, et al. Physical activity levels in three Brazilian birth cohorts as assessed with raw triaxial wrist accelerometry. Int J Epidemol. 2014;43(6):1959-68.

Hildebrand M, Hansen BH, van Hees VT, Ekelund U. Evaluation of raw acceleration sedentary thresholds in children and adults. Med Sci Sports Exerc. 2014; 46(9):1816-24.

**Appendix 2d: Fidelity assessment**

The fidelity of intervention delivery was monitored through direct observation of sessions by the Leicester Diabetes Centre staff using two instruments.

1. the STEPWISE Core Facilitator Behavioural Observation Sheet assesses the relative presence or absence of 35 behaviours in six domains: non-judgemental engagement of participants (5 Items); eliciting and responding to emotions/feelings (2 Items); facilitating reflective learning (8 Items); behavioural change, planning and goal-setting (9 items); overall group management (9 Items); other behaviours (2 items). This tool was developed especially for STEPWISE, but derived from the existing Observation Sheet used in the DESMOND Programme.
2. Second, objective Leicester Diabetes Centre staff assessed participant–educator interaction during observation visits by means of the DOT (DESMOND Observation Tool). The coder sat at the back of the room, with a CD playing in a headphone, from which a beep sounded every ten seconds, whereupon the coder recorded whether an educator or a participant was currently talking at that point. Silence, laughter or multiple conversations were classed as ‘miscellaneous’.

In self-management programme research, a link has been proposed between less facilitator talk and a more effective participant receipt of the education process, defined as a less didactic/more facilitative approach.

Further details of the tools are available on request from the authors.

**Appendix 3: Schedule of assessments**

|  | Before baseline | Baseline | Randomisation | 3 month follow-up | 12 month follow-up |
| --- | --- | --- | --- | --- | --- |
| Eligibility criteria assessed by clinical care team | ⚫ |  |  |  |  |
| Medical history |  | ⚫ |  |  |  |
| Psychiatric history |  | ⚫ |  |  |  |
| Operational Criteria Checklist for Psychotic Illness and Affective Illness^a^ |  | ⚫ |  |  |  |
| Renal function^b^ |  | ⚫ |  |  |  |
| Hepatic function^b^ |  | ⚫ |  |  |  |
| Height (to calculate body mass index) |  | ⚫ |  |  |  |
| Weight |  | ⚫ |  | ⚫ | ⚫ |
| Waist circumference |  | ⚫ |  | ⚫ | ⚫ |
| Wrist worn accelerometer up to 7 days^b^ |  | ⚫ |  | ⚫ | ⚫ |
| Adapted Dietary Instrument for Nutrition Education questionnaire |  | ⚫ |  | ⚫ | ⚫ |
| Blood Pressure (BP) |  | ⚫ |  | ⚫ | ⚫ |
| Fasting glucose^b^ |  | ⚫ |  |  | ⚫ |
| Lipid profile^b^ |  | ⚫ |  |  | ⚫ |
| Glycated haemoglobin (HbA_1c_)^b^ |  | ⚫ |  |  | ⚫ |
| EQ-5D-5L |  | ⚫ |  | ⚫ | ⚫ |
| RAND SF36 |  | ⚫ |  | ⚫ | ⚫ |
| Brief Illness Perception Questionnaire |  | ⚫ |  | ⚫ | ⚫ |
| Brief Psychiatric Rating Scale |  | ⚫ |  | ⚫ | ⚫ |
| Smoking status |  | ⚫ |  | ⚫ | ⚫ |
| Client Service Receipt Inventory |  | ⚫ |  | ⚫ | ⚫ |
| Changes in medication |  | ⚫ |  | ⚫ | ⚫ |
| Patient Health Questionnaire 9 |  | ⚫ |  | ⚫ | ⚫ |
| Use of weight loss programmes |  |  |  | ⚫ | ⚫ |
| Adverse events |  |  |  | ⚫ | ⚫ |
| Randomisation |  |  | ⚫ |  |  |
| Session Feedback |  |  |  | ⚫ | |

^a^ Completed from case note review. ^b^ Collection was permitted after randomisation where recruitment was close to start of an education course. ^c^ Intervention arm only. The follow-up window was defined as -2 to +4 weeks.

**Appendix 4: Supplementary results**

**Figure 4.1:** Individual weight change in both STEPWISE and treatment as usual groups at 3- and 12-months

**
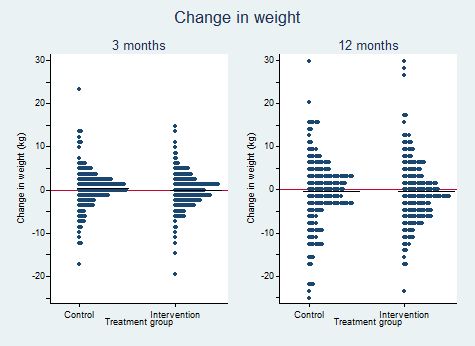
**

**Table 4.1: OPCRIT+ diagnosis of participants at baseline**

| OPCRIT+ diagnosis | Intervention (N=207) | Control (N=205) |
| --- | --- | --- |
| Schizophrenia [F20] | 37 (17.9%) | 37 (18.0%) |
| Schizo-affective disorder [F25.x] | 8 (3.9%) | 7 (3.4%) |
| Other non-organic psychosis [F28.x] | 72 (34.8%) | 69 (33.7%) |
| Affective disorder [F3X.X, F30X, F31, F32] | 11 (5.3%) | 14 (6.8%) |
| Other | 60 (29.0%) | 62 (30.2%) |
| Not met | 12 (5.8%) | 12 (5.9%) |
| Missing | 7 (3.4%) | 4 (2.0%) |

The performance of the OPCRIT+ relies on complete and accurately recorded clinical data. In many instances, there was insufficient data to form a full diagnosis using this tool. However, only 25 participants (6%) had an OPCRIT+ that would have excluded them from the trial.

Appendix table 4.2: STEPWISE average group size

|  | Mean | Median | Minimum | Maximum |
| --- | --- | --- | --- | --- |
|  |  |  |  |  |
| Group size at randomisation | 6.3 | 6 | 3 | 11 |
|  |  |  |  |  |
| Foundation courses* |  |  |  |  |
| Week 1 | 4.4 | 4 | 2 | 9 |
| Week 2 | 4.2 | 4 | 1 | 8 |
| Week 3 | 4.2 | 4 | 2 | 9 |
| Week 4 | 4.0 | 4 | 1 | 9 |
|  |  |  |  |  |
| Booster sessions* |  |  |  |  |
| Month 4 | 3.0 | 3 | 0 | 8 |
| Month 7 | 2.8 | 3 | 0 | 7 |
| Month 10 | 2.7 | 3 | 0 | 6 |

*Average number attending per course (at least 60 minutes)

**Appendix table 4.3: Attendance at external weight loss programmes**

|  | Intervention (N=207) | Control (N=205) |
| --- | --- | --- |
|  |  |  |
| 3 months |  |  |
| Followed up | 178 | 180 |
| Attended any weight loss programme | 5 (2.8%) | 10 (5.6%) |
| Slimming World | 2 | 1 |
| Weight Watchers | 0 | 3 |
| Structured programme organised by GP/care team | 1 | 3 |
| Other | 1 | 1 |
|  |  |  |
| 12 months |  |  |
| Followed up | 165 | 170 |
| Attended any weight loss programme | 19 (11.5%) | 10 (5.9%) |
| Slimming World | 8 | 3 |
| Weight Watchers | 4 | 1 |
| Structured programme organised by GP/care team | 3 | 4 |
| Other | 1 | 4 |

**Appendix table 4.4: Dietary intake as assessed by adapted Dietary Instrument for Nutrition Education (DINE) questionnaire at baseline, 3-month and 12-month follow-up visits.**

|  | Baseline | | 3-month | | 12-month | |
| --- | --- | --- | --- | --- | --- | --- |
|  | Intervention (N=207) | Control (N=205) | Intervention (N=178) | Control (N=180) | Intervention (N=164) | Control (N=171) |
| **Dietary Fibre** |  |  |  |  |  |  |
| Mean (s.d.) | 27.6 (11.3) | 28.3 (12.6) | 28.2 (12.0) | 28.0 (12.4) | 28.6 (12.1) | 28.1 (10.6) |
| Median (i.q.r.) | 26.0 (19.0, 34.0) | 26.0 (20.0, 36.0) | 26.0 (19.0, 36.0) | 26.5 (19.5, 36.0) | 28.0 (20.0, 35.0) | 28.0 (21.0, 36.0) |
| Low | 122 (58.9%) | 120 (58.5%) | 101 (56.7%) | 103 (57.2%) | 90 (54.5%) | 93 (54.1%) |
| Medium | 55 (26.6%) | 50 (24.4%) | 41 (23.0%) | 53 (29.4%) | 52 (31.5%) | 61 (35.5%) |
| High | 30 (14.5%) | 35 (17.1%) | 36 (20.2%) | 24 (13.3%) | 23 (13.9%) | 18 (10.5%) |
| **Fat Intake** |  |  |  |  |  |  |
| Mean (s.d.) | 31.5 (11.0) | 32.2 (11.9) | 30.1 (11.4) | 30.2 (11.6) | 30.6 (12.7) | 30.6 (11.6) |
| Median (i.q.r.) | 31.0 (23.0, 39.0) | 30.0 (23.0, 39.0) | 28.0 (22.0, 36.0) | 29.0 (21.0, 37.0) | 28.0 (22.0, 37.0) | 30.0 (22.0, 37.5) |
| Low | 96 (46.4%) | 96 (46.8%) | 99 (55.6%) | 97 (53.9%) | 90 (54.5%) | 80 (46.5%) |
| Medium | 65 (31.4%) | 63 (30.7%) | 49 (27.5%) | 52 (28.9%) | 41 (24.8%) | 62 (36.0%) |
| High | 46 (22.2%) | 46 (22.4%) | 30 (16.9%) | 31 (17.2%) | 34 (20.6%) | 30 (17.4%) |
| **Unsaturated fat intake** |  |  |  |  |  |  |
| Mean (s.d.) | 9.5 (1.6) | 9.2 (1.9) | 9.6 (1.9) | 9.3 (1.9) | 9.7 (1.8) | 9.4 (1.9) |
| Median (i.q.r.) | 10.0 (9.0, 11.0) | 9.0 (8.0, 11.0) | 10.0 (9.0, 11.0) | 9.0 (9.0, 11.0) | 10.0 (9.0, 11.0) | 10.0 (9.0, 11.0) |
| Low | 6 (2.9%) | 12 (5.9%) | 6 (3.4%) | 9 (5.0%) | 3 (1.8%) | 7 (4.1%) |
| Medium | 93 (44.9%) | 94 (45.9%) | 65 (36.5%) | 81 (45.0%) | 68 (41.2%) | 78 (45.3%) |
| High | 108 (52.2%) | 98 (47.8%) | 107 (60.1%) | 89 (49.4%) | 93 (56.4%) | 86 (50.0%) |
| **Daily sugar intake from drinks** |  |  |  |  |  |  |
| Mean (s.d.) | 76.2 (73.1) | 85.4 (85.1) | 70.9 (78.9) | 74.2 (81.0) | 71.8 (90.6) | 66.3 (77.9) |
| Median (i.q.r.) | 53.0 (25.0, 102.0) | 56.0 (29.0, 104.0) | 49.0 (19.0, 86.0) | 50.0 (22.0, 93.0) | 37.0 (12.0, 90.0) | 41.0 (19.0, 91.0) |
| Weekly alcohol intake |  |  |  |  |  |  |
| Mean (s.d.) | 4.6 (12.6) | 3.0 (7.4) | 3.5 (10.3) | 3.5 (12.8) | 3.7 (9.9) | 4.5 (12.2) |
| Median (i.q.r.) | 0.5 (0.0, 3.5) | 0.5 (0.0, 3.0) | 0.0 (0.0, 2.5) | 0.0 (0.0, 2.5) | 0.5 (0.0, 2.5) | 0.0 (0.0, 3.5) |
| **Weekly alcohol intake (units)** |  |  |  |  |  |  |
| Mean (s.d.) | 4.6 (12.6) | 3.0 (7.4) | 3.5 (10.3) | 3.5 (12.8) | 3.7 (9.9) | 4.5 (12.2) |
| Median (i.q.r.) | 0.5 (0.0, 3.5) | 0.5 (0.0, 3.0) | 0.0 (0.0, 2.5) | 0.0 (0.0, 2.5) | 0.5 (0.0, 2.5) | 0.0 (0.0, 3.5) |

**Appendix table 4.5: Smoking Status at baseline, 3-month and 12-month follow-up visits.**

|  | Baseline | | 3-month | | 12-month | |
| --- | --- | --- | --- | --- | --- | --- |
|  | Intervention (N=207) | Control (N=205) | Intervention (N=178) | Control (N=180) | Intervention (N=166) | Control (N=172) |
|  |  |  |  |  |  |  |
| **Current smoker** | 98 (47.3%) | 108 (52.7%) | 85 (47.8%) | 96 (53.3%) | 75 (45.2%) | 88 (51.2%) |
| Light smoker | 18 (8.7%) | 20 (9.8%) | 13 (7.3%) | 19 (10.6%) | 17 (10.2%) | 13 (7.6%) |
| Moderate smoker | 37 (17.9%) | 32 (15.6%) | 28 (15.7%) | 31 (17.2%) | 29 (17.5%) | 27 (15.7%) |
| Heavy smoker | 43 (20.8%) | 55 (26.8%) | 44 (24.7%) | 46 (25.6%) | 29 (17.5%) | 48 (27.9%) |
| Amount not reported | 0 (0.0%) | 1 (0.5%) |  |  |  |  |
|  |  |  |  |  |  |  |
| **Offered help to stop smoking** | 89 (47.3%) | 90 (47.1%) | 78 (47.0%) | 71 (42.5%) | 64 (45.1%) | 63 (42.9%) |
| Brief intervention | 38 (20.2%) | 53 (27.7%) | 39 (23.5%) | 39 (23.4%) | 27 (19.0%) | 27 (18.4%) |
| Nicotine replacement | 58 (30.9%) | 61 (31.9%) | 55 (33.1%) | 54 (32.3%) | 47 (33.1%) | 42 (28.6%) |
| Drug treatment | 3 (1.6%) | 4 (2.1%) | 4 (2.4%) | 2 (1.2%) | 1 (0.7%) | 3 (2.0%) |
| Electronic cigarettes/vape | 5 (2.7%) | 3 (1.6%) | 5 (3.0%) | 1 (0.6%) | 3 (2.1%) | 3 (2.0%) |
| Other types | 8 (4.3%) | 6 (3.1%) | 2 (1.2%) | 2 (1.2%) | 6 (4.2%) | 2 (1.4%) |
|  |  |  |  |  |  |  |
| **Currently using therapy to stop smoking** | 24 (12.8%) | 25 (13.1%) | 21 (12.7%) | 19 (11.4%) | 22 (15.4%) | 11 (7.5%) |
| Nicotine replacement | 15 (8.0%) | 013 (6.8%) | 11 (6.6%) | 13 (7.8%) | 12 (8.4%) | 6 (4.1%) |
| Drug treatment | 0 | 0 | 0 | 0 | 0 | 0 |
| Electronic cigarettes/vape | 10 (5.3%) | 12 (6.3%) | 10 (6.0%) | 6 (3.6%) | 10 (7.0%) | 4 (2.7%) |
| Other types | 9 (4.8%) | 12 (6.3%) | 9 (5.4%) | 6 (3.6%) | 9 (6.3%) | 5 (3.4%) |

**Appendix table 4.6: RAND-SF36 at baseline, 3-month and 12-month follow-up visits Baseline characteristics. Data are mean (SD). Statistical analysis is on the basis of intention to treat**

|  | Baseline | | 3-month | | | | 12-month | | | |
| --- | --- | --- | --- | --- | --- | --- | --- | --- | --- | --- |
|  | Intervention (N=207) | Control (N=205) | | Intervention (N=178) | Control (N=180) | Difference between Intervention and Control | | Intervention (N=167) | Control (N=173) | Difference between Intervention and Control |
|  |  |  | |  |  |  | |  |  |  |
| Physical Functioning | 72.8 (25.6) | 71.9 (24.6) | | 76.9 (23.1) | 70.9 (26.2) | 4.4 (0.9,7.9) | | 76.5 (25.6) | 70.9 (26.8) | 4.1 (0.1,8.1) |
| Role limitations due to physical health | 54.6 (42.0) | 54.2 (41.0) | | 63.8 (39.7) | 60.2 (41.3) | 3.3 (-4.8,11.4) | | 65.9 (41.8) | 60.8 (40.7) | 4.8 (-3.0,12.6) |
| Role limitations due to emotional problems | 46.2 (42.6) | 44.0 (42.3) | | 52.2 (43.8) | 50.1 (44.0) | 1.3 (-7.0,9.6) | | 58.5 (43.9) | 52.5 (45.4) | 5.1 (-3.6,13.7) |
| Energy/fatigue score | 43.2 (21.8) | 40.5 (23.1) | | 44.9 (21.6) | 42.8 (23.6) | -0.4 (-3.5,2.7) | | 44.9 (22.2) | 45.0 (24.6) | -2.5 (-6.2,1.1) |
| Emotional well-being | 58.3 (21.4) | 56.4 (23.9) | | 58.8 (22.3) | 57.0 (25.3) | -0.1 (-3.4,3.3) | | 58.8 (23.4) | 62.2 (23.4) | -5.2 (-8.9,-1.5) |
| Social functioning | 60.5 (29.1) | 61.2 (28.7) | | 64.5 (29.6) | 62.7 (31.5) | 2.8 (-2.7,8.4) | | 66.1 (30.0) | 65.7 (29.5) | 1.5 (-4.3,7.2) |
| Bodily Pain | 71.6 (28.8) | 74.8 (27.4) | | 75.2 (25.5) | 71.3 (28.4) | 5.5 (0.9,10.1) | | 71.6 (30.7) | 70.6 (29.1) | 2.1 (-2.3,6.5) |
| General Health | 45.0 (20.3) | 44.8 (20.7) | | 48.0 (21.8) | 46.8 (20.3) | -0.3 (-3.4,2.8) | | 49.8 (23.1) | 46.8 (21.4) | 2.2 (-1.3,5.6) |
| Health Change | 57.4 (30.2) | 57.4 (29.0) | | 62.1 (26.7) | 58.6 (28.9) |  | | 62.5 (25.3) | 63.4 (26.7) |  |

**Appendix table 4.7: Condition perception as measured by the Brief Illness Perception Questionnaire (B-IPQ) at baseline, 3-month and 12-month follow-up visits Baseline characteristics. Data are mean (SD). Statistical analysis is on the basis of intention to treat**

|  | Baseline | | | 3-month | | | 12-month | | |
| --- | --- | --- | --- | --- | --- | --- | --- | --- | --- |
|  | Intervention (N=207) | Control (N=205) | Intervention (N=178) | | Control (N=180) | Intervention (N=164) | | Control (N=170) |  |
|  |  |  |  | |  |  | |  |  |
| Overall | 5.5 (1.5) | 5.5 (1.7) | 5.0 (1.7) | | 5.3 (1.7) | 5.0 (1.9) | | 5.0 (1.7) |  |
| How much does your weight problem affect your life? | 6.1 (2.7) | 6.2 (2.8) | 5.9 (2.8) | | 6.1 (2.9) | 5.9 (3.0) | | 5.7 (2.9) |  |
| How long do you think your weight problem will continue? | 6.1 (2.5) | 6.0 (2.7) | 5.9 (2.4) | | 5.9 (2.6) | 5.7 (2.7) | | 5.6 (2.9) |  |
| How much control do you feel you have over your weight problem? | 4.4 (2.6) | 4.1 (2.9) | 5.4 (2.5) | | 4.7 (2.8) | 5.4 (2.6) | | 4.9 (3.0) |  |
| How much do you think lifestyle programmes can help your weight problem? | 7.2 (2.1) | 6.7 (2.6) | 6.8 (2.7) | | 5.9 (2.7) | 6.5 (2.7) | | 6.0 (3.0) |  |
| How much do you experience symptoms from your weight problem? | 5.4 (3.0) | 5.2 (3.1) | 4.6 (3.0) | | 4.7 (3.2) | 4.9 (3.2) | | 4.5 (3.2) |  |
| How concerned are you about your weight problem? | 7.6 (2.6) | 7.4 (2.7) | 7.0 (2.9) | | 7.0 (2.8) | 6.8 (2.8) | | 6.4 (3.2) |  |
| How well do you feel you understand your weight problem? | 6.4 (2.8) | 6.7 (2.8) | 7.2 (2.6) | | 6.8 (2.8) | 7.1 (2.7) | | 6.8 (2.9) |  |
| How much does your weight problem affect you emotionally? | 6.1 (2.9) | 6.1 (3.4) | 5.5 (3.3) | | 6.0 (3.4) | 5.3 (3.2) | | 5.2 (3.2) |  |

Appendix table 4.8a. Session Feedback questions and responses overall (n=703 for each question)

|  | **Responses (%)** | | | | |
| --- | --- | --- | --- | --- | --- |
|  | **1**  **Strongly agree** | **2** | **3**  **Neither agree nor disagree** | **4** | **5**  **Strongly disagree** |
| The facilitator listened to me | 77.1 | 12.5 | 2.0 | 2.0 | 6.4 |
| I understood what we talked about | 74.4 | 13.9 | 3.0 | 2.3 | 6.4 |
| I found what we talked about useful | 71.8 | 17.1 | 2.7 | 2.6 | 5.8 |
| I felt the facilitator understood the challenges I face | 66.1 | 20.3 | 5.1 | 3.0 | 5.4 |
| What we talked about made sense to me | 76.7 | 12.4 | 2.4 | 3.3 | 5.3 |
| Overall the session met my needs | 67.3 | 19.9 | 4.7 | 3.1 | 5.0 |

Appendix table 4.8b. Session feedback scores (n = 703) by Centre

|  | Forms returned | Total Score  Mean (sd) | Total score  Median | IQR | Score 6  (most positive)  % | Score >24  (most negative)  % |
| --- | --- | --- | --- | --- | --- | --- |
| Sheffield | 51 | 7.9 (4.3) | 7 | 6 - 8 | 47.1 | 3.9 |
| Leeds & York | 39 | 8.1 (4.2) | 6 | 6 - 10 | 64.1 | 2.6 |
| Bradford | 70 | 10.6 (8.5) | 6 | 6 - 11 | 58.6 | 15.7 |
| Manchester | 84 | 7.7 (3.6) | 6 | 6 - 9 | 61.9 | 1.2 |
| South London | 61 | 10.1 (7.1) | 6 | 6 - 11 | 54.1 | 8.2 |
| Sussex | 105 | 9.6 (5.8) | 7 | 6 - 11 | 45.7 | 5.7 |
| Southern Health | 116 | 9.2 (6.3) | 6 | 6 - 9 | 57.8 | 7.8 |
| Devon | 69 | 9.7 (7.0) | 6 | 6 - 10 | 56.5 | 10.1 |
| Somerset | 26 | 7.6 (3.0) | 6 | 6 - 8 | 65.4 | 0 |
| Cornwall | 82 | 9.9 (6.1) | 8 | 6 - 11 | 41.5 | 7.3 |
| **Overall*** | **703** | **9.2 (6.1)** | **6** | **6 - 10** | **54.1** | **6.8** |

*all centres have at least 75% of forms scoring below 12 (equivalent to scoring 2 for each statement).

Appendix table 4.9. Summary of adverse events

|  | Intervention (N=207) | | Control (N=205) | |
| --- | --- | --- | --- | --- |
|  | No. Events | No (%) participants | No. Events | No (%) participants |
| Any AE | 46 | 37 (17.9%) | 34 | 26 (12.7%) |
| Psychiatric hospitalisation | 23 | 20 (9.7%) | 17 | 16 (7.8%) |
| Self-harm | 0 |  | 1 | 1 (0.55%) |
| Suicide attempt | 2 | 2 (1.0%) | 2 | 2 (1.0%) |
| Hospitalisation (not mental health related) | 13 | 11 (5.3%) | 11 | 10 (4.9%) |
| Death | 3 | 3 (1.4%) | 0 |  |
| Skin reaction to accelerometry | 4 | 4 (1.9%) | 0 |  |
| Other | 1 | 1 (0.5%) | 3 | 3 (1.5%) |

A further death in the intervention group was reported 37 days after trial completion. The causes of death were: pulmonary embolism likely secondary to a ruptured Achilles tendon; myocardial infarction; diabetic ketoacidosis; left ventricular hypertrophy. None of the deaths was considered to be a result of the intervention.

Appendix table 4.10. Fidelity ratings for session sections observed by centre

| **Centre** | **Group**  **Sessions Observed (N)** | **‘DOT’ scored**  **Session Sections**  **(N)** | **% Facilitator spoke**  **Mean (SD)** | **% Facilitator spoke**  **Median (Range)** | **Facilitator Behaviour scores**  **Session Sections**  **(N)** | **% Positive (‘Left’)**  **Mean (SD)** | **% Positive (‘Left’)**  **Median (Range)** | **% Negative (‘Right’)**  **Mean (SD)** | **% Negative (‘Right’)**  **Median (Range)** |
| --- | --- | --- | --- | --- | --- | --- | --- | --- | --- |
| Bradford | 2 | 8 | 46.6 (6.85) | 45.5  (39.0 – 57.0) | 8 | 46.4 (12.19) | 47.1  (25.7 – 62.9) | 26.8 (8.21) | 28.6  (14.3 -37.1) |
| Cornwall | 2 | 8 | 63.2 (9.40) | 63.8  (50.0 – 78.0) | 8 | 31.8 (13.18) | 34.3  (14.3 – 54.3) | 38.9 (13.9) | 35.7  (20.0 – 62.9) |
| Devon | 2 | 7 | 36.8 (6.39) | 37.0  (28.0 - 44.0) | 7 | 59.6 (6.69) | 57.1  (54.3 – 71.4) | 3.7 (2.16) | 2.9  (0 – 5.7) |
| Leeds & York | 6 | 22 | 50.0 (13.07) | 52.5  (27.0 – 78.4) | 24 | 57.5 (10.23) | 57.1  (37.1 – 77.1) | 21.0 (11.54) | 20.0  (2.9 – 40.0) |
| Manchester | 2 | 8 | 41.6 (8.26) | 45.5  (29.0 – 50.0) | 8 | 64.6 (17.67) | 65.7  (42.9 – 85.7) | 7.1 (11.7) | 0.0  (0 – 28.6) |
| Sheffield | 1 | 4 | 42.5 (5.22) | 42.3  (36.4 – 48.8) | 4 | 63.6 (16.88) | 65.7  (42.9 – 80.0) | 20.7 (15.54) | 15.7  (8.6 – 42.9) |
| Somerset | 1 | 4 | 44.0 (8.83) | 45.5  (32.0 – 53.0) | 4 | 64.3 (6.80) | 61.4  (60.0 – 74.3) | 15.0 (4.88) | 15.7  (8.6 – 20.0) |
| Southern Health | 2 | 8 | 50.8 (8.17) | 49.0  (41.0 – 66.0) | 8 | 45.4 (11.11) | 45.7  (28.6 – 62.9) | 41.4 (14.57) | 40.0  (14.3 – 60.0) |
| South London | 6 | 23 | 49.3(12.96) | 48.1  (25.0 – 75.0) | 23 | 48.1 (14.17) | 45.7  (20.0– 71.4) | 31.4 (14.47) | 28.6  (11.4 – 65.7) |
| Sussex | 6 | 24 | 44.5 (12.93) | 44.5  (22.0 – 65.0) | 23 | 61.5 (12.24) | 57.1  (34.3 – 85.7) | 20.7 (12.4) | 20.0  (0 – 40.0) |
| Overall | 30 | 116 | 47.6 (12.26) | 47.4  (22.0 – 78.4) | 117 | 54.1 (14.97) | 54.3  (14.3 – 85.7) | 23.8 (15.45) | 22.9  (0 – 65.7) |
